# Predicting starch content in cassava fresh roots using near-infrared spectroscopy

**DOI:** 10.3389/fpls.2022.990250

**Published:** 2022-11-08

**Authors:** Edwige Gaby Nkouaya Mbanjo, Jenna Hershberger, Prasad Peteti, Afolabi Agbona, Andrew Ikpan, Kayode Ogunpaimo, Siraj Ismail Kayondo, Racheal Smart Abioye, Kehinde Nafiu, Emmanuel Oladeji Alamu, Michael Adesokan, Busie Maziya-Dixon, Elizabeth Parkes, Peter Kulakow, Michael A. Gore, Chiedozie Egesi, Ismail Yusuf Rabbi

**Affiliations:** ^1^ International Institute of Tropical Agriculture (IITA), Ibadan, Oyo State, Nigeria; ^2^ Department of Plant and Environmental Sciences, Pee Dee Research and Education Center, Clemson University, Florence, SC, United States; ^3^ Molecular & Environmental Plant Sciences, Texas A&M University, College Station, TX, United States; ^4^ Plant Breeding and Genetics Section, School of Integrative Plant Science, Cornell University, Ithaca, NY, United States; ^5^ National Root Crops Research Institute (NRCRI), Umuahia, Nigeria

**Keywords:** cassava, starch, NIRS, spectrophotometers, SCiO, spectra, heritability

## Abstract

The cassava starch market is promising in sub-Saharan Africa and increasing rapidly due to the numerous uses of starch in food industries. More accurate, high-throughput, and cost-effective phenotyping approaches could hasten the development of cassava varieties with high starch content to meet the growing market demand. This study investigated the effectiveness of a pocket-sized SCiO™ molecular sensor (SCiO) (740−1070 nm) to predict starch content in freshly ground cassava roots. A set of 344 unique genotypes from 11 field trials were evaluated. The predictive ability of individual trials was compared using partial least squares regression (PLSR). The 11 trials were aggregated to capture more variability, and the performance of the combined data was evaluated using two additional algorithms, random forest (RF) and support vector machine (SVM). The effect of pretreatment on model performance was examined. The predictive ability of SCiO was compared to that of two commercially available near-infrared (NIR) spectrometers, the portable ASD QualitySpec^®^ Trek (QST) (350−2500 nm) and the benchtop FOSS XDS Rapid Content™ Analyzer (BT) (400−2490 nm). The heritability of NIR spectra was investigated, and important spectral wavelengths were identified. Model performance varied across trials and was related to the amount of genetic diversity captured in the trial. Regardless of the chemometric approach, a satisfactory and consistent estimate of starch content was obtained across pretreatments with the SCiO (correlation between the predicted and the observed test set, (*R^2^*_P_): 0.84−0.90; ratio of performance deviation (RPD): 2.49−3.11, ratio of performance to interquartile distance (RPIQ): 3.24−4.08, concordance correlation coefficient (CCC): 0.91−0.94). While PLSR and SVM showed comparable prediction abilities, the RF model yielded the lowest performance. The heritability of the 331 NIRS spectra varied across trials and spectral regions but was highest (*H^2^* > 0.5) between 871−1070 nm in most trials. Important wavelengths corresponding to absorption bands associated with starch and water were identified from 815 to 980 nm. Despite its limited spectral range, SCiO provided satisfactory prediction, as did BT, whereas QST showed less optimal calibration models. The SCiO spectrometer may be a cost-effective solution for phenotyping the starch content of fresh roots in resource-limited cassava breeding programs.

## Introduction

The global starch market is experiencing increased demand, with an estimated value of US$ 51.5 billion in 2021 and a projected value of US$70.5 billion by 2027^1^. Starch is a polysaccharide that plants produce as a carbohydrate reserve. Approximately 54% of the starches produced globally are used for food. In comparison, 46% are used in non-food products such as textiles, pharmaceuticals, pulp and paper, adhesives for packing industries, and cosmetics manufacturing (Omojola, 2013; Desta and Tigabu, 2018; Raji, 2020). Cassava starch, with its excellent characteristics and favorable physicochemical and functional properties, could be an alternative source of starch in a market traditionally dominated by cereal and potato starches (Oladunmoye et al., 2014; Spencer and Ezedinma, 2017; Chisenga et al., 2019).

Cassava (*Manihot esculanta* Crantz) is a climate-resilient crop owing to its tolerance to drought, poor soils, and wide adaptability to various climate and cropping systems. It is also a poverty alleviating crop, primarily grown for human consumption. Cassava is gradually evolving into an industrial crop (Chisenga et al., 2019). The significantly increased demand for starch and starch-based products combined with the inability of traditional exporters to meet market demand provides new opportunities for the crop in sub-Saharan Africa. Cassava has the potential to contribute to income, social progress and development, and economic growth in countries that produce it (Dada, 2016). Therefore, cassava production in sub-Saharan Africa should increase to meet rising market demand. In the face of resource depletion, land scarcity, urbanization, and rapid population growth, increasing starch production by expanding cassava cultivation land areas is not a sustainable solution. An alternative solution to close the demand gap is developing high starch content cassava varieties.

Breeding efforts for cassava varieties with high starch content could be accelerated by developing high-throughput phenotyping tools that can rapidly and precisely assess numerous genotypes at an early stage. Phenotyping remains one of the major limitations hindering the power of genetic analysis of key traits and accurate selection of superior genotypes at all stages of the breeding process (Cobb et al., 2013; Reynolds et al., 2020). Several high-throughput, non-invasive phenotyping technologies, such as image analysis (Baek et al., 2020), satellite imaging, and remote sensing with unoccupied aerial vehicles (Chawade et al., 2019), have recently been developed, opening up new opportunities in breeding. In cassava, spectroscopy-based approaches that use near-infrared (NIR) regions of the electromagnetic spectrum have shown promise for the rapid estimation of key traits (Sánchez et al., 2014; Abincha et al., 2021; Hershberger et al., 2022). Near-infrared technology could replace the laborious and time-consuming approach currently used for root starch content quantification.

NIR spectroscopy studies the spectral properties of an object when exposed to electromagnetic radiation. Light from the NIR region may be absorbed, reflected, or transmitted. The resulting spectrum is associated with molecular vibrational excitation caused by overtones and a combination of a specific set of chemicals bound from within a molecule (Ozaki et al., 2020; Beć et al., 2021). The NIR region is further classified into three sub-categories: region I (800−1200 nm), also known as the Herschel region, region II (1200−1800 nm), and region III (1800−2500 nm). Technological advancement has fostered the development of miniaturized NIR devices with limited spectral ranges but offering significant advantages in terms of price and portability over traditional spectrometers with full spectral ranges. However, these advantages may come at the expense of accuracy and robustness. As a result, such devices must be assessed for analytical performance and model reliability (Ozaki et al., 2020; Beć et al., 2021).

Our study investigates the potential of a miniaturized SCiO™ spectrometer as an alternative phenotyping method for determining starch content in fresh cassava roots. Cassava clones (hereafter referred to as genotypes) from 11 trials were harvested and their starch was extracted and quantified. Using three chemometric modeling approaches, random forest (RF), support vector machine (SVM), and partial least squares regression (PLSR), the relationship between reference values and NIR spectra collected with the SCiO™ molecular sensor was established. The heritability of individual wavelengths was investigated to determine the degree to which variation for a wavelength is due to genetic variation among genotypes. The most effective wavelengths in this experiment for predicting starch content were identified using variable importance analysis. The SCiO™ sensor’s performance was compared to two different NIRS instruments: the portable ASD QualitySpec^®^ Trek and the benchtop FOSS XDS Rapid Content™ Analyzer. It was established that SCiO™ could be a rapid analytic tool for measuring starch content, allowing breeders to screen large populations at an early stage.

## Materials and methods

### Plant material

The set of genotypes used in this study was composed of genotypes from preliminary yield trials (PYTs), advanced yield trials (AYTs), uniform yield trials, regional nationally coordinated research program (NCRP) trials, and genomic selection (GS) cycles (**Table 1**). These trials were established across three locations in Nigeria (Ikenne, Ibadan, and Ago-Owu) in the 2019 and 2020 rainy seasons. Mature roots were harvested 12 months after planting (MAP). In total, 344 unique genotypes from 11 field trials were evaluated. These included an early-stage evaluation (PYT Trial A) with 174 unique genotypes planted in one environment; three late-stage evaluation trials (UYT and AYT Trials J, H, I) with between 36 and 40 genotypes planted in two replicates in a single environment; a pre-release trial (NCRP Trials E, F) with 18 unique genotypes planted in three replicates across two environments; and trials of two germplasm collections maintained by the IITA cassava breeding program. The first collection comprised a popular landrace and improved varieties widely cultivated in Nigeria with 33 unique genotypes and was planted in two replicates across three environments (Trial C, D, G). The second collection, which comprised 52 unique genotypes, was planted in replicated PYTs across two environments (Trial B and K). This second collection (also considered a “core collection”) was selected from a large pool of historically important breeding lines from IITA (Okechukwu and Dixon, 2008) and contains substantial variation for important agronomic traits, including fresh root yield and starch content.

**Table 1:** Cassava breeding field trial metadata and summary statistics for root starch content.

Cassava base trial name	Abbreviated trial name	Date of planting	Date of harvest	Trial type^a^	Trial design^b^	Location	Min^b^	Max^c^	SD^d^	CV^e^	Plots used	Unique genotypes
19.GS.C4B.PYT.500.IK	Trial A	4 Aug.2019	27.Oct. 2020	PYT	RCBD	Ikenne	18.7	41.6	3.45	0.11	261	174
19.CASS.PYT.52.IK	Trial B	25 June 2019	27 Oct.2020	PYT	RCBD	Ikenne	4.1	38.8	8.07	0.37	97	50
19.CMSSurveyVarieties.AYT33.IK	Trial C	10 May 2019	23 Apr. 2020	AYT	Alpha-Lattice	Ikenne	19.9	37.8	4.21	0.15	65	32
19.CMSSurveyVarieties.AYT.33.IB	Trial D	29 Apr 2019	20 Apr. 2020	AYT	Alpha-Lattice	Ibadan	10.1	30.3	5.01	0.25	52	31
19NCRPAG	Trial E	2 July 2019	27 July 2020	NCRP	Alpha-Lattice	Ago-Owu	18.6	35.3	3.6	0.14	36	18
19NCRPIK	Trial F	4 Aug 2019	28 July 2020	NCRP	Alpha-Lattice	Ikenne	20.2	37	3.77	0.13	36	18
20.CMSSurveyVarieties.AYT.33.IB	Trial G	24 Apr 2020	24 Apr. 2021	AYT	Alpha-Lattice	Ibadan	14.3	31.35	4.34	0.18	65	33
20.GS.C2.UYT.36.SetA.IB	Trial H	15 May 2020	15 May 2021	UYT	Alpha-Lattice	Ibadan	14.5	31.8	3.57	0.14	71	36
20.GS.C2.UYT.36.SetB.IB	Trial I	15 May 2020	18 May 2021	UYT	RCBD	Ibadan	17.55	31.65	2.62	0.1	72	36
20.GS.C4B.AYT.40.IB	Trial J	10 June 2020	30 July 2021	AYT	RCBD	Ibadan	13.65	27.85	2.94	0.13	80	40
20.CASS.PYT.52.IK	Trial K	12 July 2020	17 March 2021	PYT	RCBD	Ikenne	10.1	34.6	6.59	0.3	88	50
							4.10	41.6	6.23	0.24	921	518/344

a, minimum; b, maximum, c, standard deviation; d, coefficient of variation.

a AYT, Advanced yield trial; PYT, Preliminary yield trial; UYT, Uniform yield trial; NCRP, National coordinate research program.

b RCBD, randomized complete block design.

c Min, Minimum value; Max, Maximum value.

d Standard deviation.

e Coefficient of variation.

### Reference data measurement

Six healthy storage roots of varying sizes were randomly selected from each plot to ensure representativeness. Selected roots were free of defects such as decomposition, disease, and bruises. These roots were harvested, placed in labeled sampling bags, and immediately taken to the laboratory for starch extraction with the protocol adapted from Matsumoto et al. (2021). Briefly, roots were washed and peeled, and the proximal and distal ends of each root were removed. The top, middle, and bottom sides of each selected root were shredded with a hand grater (3-mm hole diameter). All shredded roots from each plot were mixed together. The starch of individual genotypes was extracted using a wet-milling approach with 3 L of water. One hundred grams of the mixed, shredded tissue was milled with 200 mL of water for one minute with two-second breaks. The slurry was filtered through a sieve with a mesh size of 180 μm. This filtering process was repeated until the residue turned pale white, at which point the remaining water was added to the precipitate. The precipitate was left at room temperature for three hours to allow the starch granules to settle. The supernatant was slowly decanted, and the sediment (starch) was air-dried for 72 hours at room temperature before being oven-dried for 24 hours at 40°C. The RSC, expressed as a percentage of fresh root yield, was calculated by weighing the dry sediments. The amount of starch was determined using the following equation:


$$RSC\;(\%)=\frac{DSM}{FM}\times 100$$

Where dry starch mass (DSM) is the weight of starch extracted from a known weight of the root matter and fresh root mass (FM) is the known weight of the root matter.

### Spectra acquisition

NIR spectra were acquired primarily using a pocket-sized SCiO™ (SCiO) molecular sensor (Consumer physics, Tel Aviv, Israel) that collected spectral information from 740 to 1070 nm with a resolution of 1 nm. The SCiO sensor was synced with a tablet *via* Bluetooth, enabling communication between the two devices for digital data transfer from the SCiO sensor to the SCiO cloud *via* the SCiO smartphone application. The sensor was calibrated before sample capture using a built-in reference standard in the SCiO case. The thoroughly mixed, shredded cassava roots were placed in quartz cell glasses. The SCiO optical shade was connected to the sensor and placed on the top of the cell quartz with the optical head facing down. The light source illuminated the samples and the reflected lighted captured by the detector was uploaded to the online SCiO cloud database. Each genotype was measured in three technical replicates (i.e., three independent tissue samples), and each sample was scanned three times in different positions by rotating the quartz cell glass. The spectra were downloaded as comma-separated value files from the SCiO cloud database. The various repeated scans per sample were averaged and the averaged spectrum was used for further analyses.

### Reference data analysis

Descriptive statistics for each trait [minimum and maximum values, coefficient of variation (CV), and standard deviation (SD)] were obtained using the R package pastecs (Grosjean et al., 2018). Boxplots were used to visualize starch variation in each trial. Significant differences (*P*< 0.05) between the trials were estimated using the Kruskal-Wallis rank test.

### Spectra data analysis

The raw spectra were used to classify cassava genotypes into homogeneous groups using principal component analyses (PCA). This analysis was performed using the R package factorMineR (Lê et al., 2008). The PCA plot was visually inspected to identify extreme values, and the two genotypes that deviated from most data were removed. Model development and validation were performed using the R package waves version 0.2.4 (Hershberger et al., 2021). Twelve combinations of mathematical pretreatments, standard normal variate (SNV), first derivative (D1), second derivative (D2), standard normal variate and first derivative (SNV1D), standard normal variate and second derivative (SNV2D), Savitzky-Golay filter (SG), standard normal variate and Savitzky-Golay filter (SNVSG), gap-segment derivative (window size = 11) (SGD1), Savitzky-Golay filter first derivative (window size = 5) (SG.D1W5), Savitzky-Golay filter and first derivative (window size = 11) (SG.D1W11), Savitzky-Golay filter and second derivative (window size = 5) (SG.D2W5), and Savitzky-Golay filter and second derivative (window size = 11) (SG.D2W11), were implemented within the waves R package version 0.2.4 (Hershberger et al., 2021) to minimize the effect of uncontrolled covariates (scattering effects, particle size, variation in the light path, etc.), remove noise from NIR spectra, correct non-linear trends and additive and/or multiplicative effects in the spectrum, and enable a thorough search for optimum prediction. The Mahalanobis distance of each spectrum was calculated, and outliers were removed based on Mahalanobis distance > 3. Individual trials were modeled using PLSR. When data from all 11 trials were combined, two other modeling approaches, RF and SVM with a radial kernel, were evaluated. The genotypes were divided into two sets for internal cross-validation, one for calibration (training set) and one for validation (test set). The calibration set was chosen randomly and accounted for 70% of the total genotypes, while the test set accounted for 30% of the total genotypes. Five-fold cross-validation was used to identify the model with the best prediction ability. This process was repeated 50 times (niter = 50). Several statistical parameters, including the squared Pearson’s correlation between predicted and observed values in the test set (*R^2^*_p_), the coefficient of determination extracted from the PLSR model (*R^2^*_CV_), and the root mean squared error of prediction as calculated using predicted and observed values from the test set (RMSE_P_) were used to assess the model’s goodness of fit. Other parameters included the root mean square error of cross validation extracted from the PLSR model (RMSE_CV_), the ratio of performance deviation (RPD), standard error of prediction (SEP), ratio of performance to interquartile distance (RPIQ), and Lin’s concordance correlation (CCC).

Four additional cross-validation schemes that mimic scenarios commonly encountered by plant breeders (CV2, CV1, CV0, and CV00) were applied across the 11 trials tested. Each trial was treated as an independent environment, as described by Jarquín et al. (2017). For CV2 (tested genotypes in tested environments), 30% of the genotypes from a given trial made up the test set. All remaining genotypes and all genotypes from other trials were combined to form the training set. The test sets for CV1 (untested genotypes in tested environments) were the same as for CV2; however, genotypes present in the test set were entirely removed from the training set. Each trial underwent 50 iterations of training, each with a different random sample of genotypes in the test set. For CV0 (tested genotypes in untested environments), an entire trial was included as the test set. All other trials, regardless of whether they contained genotypes represented in the test set trial, constituted the training set. CV00 (untested genotypes in untested environments) followed the same procedure as CV0, but all test set genotypes were removed from the training set prior to model training. For CV0 and CV00, only a single iteration was performed (Hershberger et al., 2022).

### Variable importance and heritability estimate

RF and PLSR models were used to assess the significance of each wavelength in predicting root starch content by calculating variable importance for each wavelength. The possibility of heritable variation along the spectra was investigated. The heritability of root starch content was also evaluated for each trial. Variance components were estimated for both scenarios using a mixed linear model and the R package lme4 (Bates et al., 2015). The trial design was used to define the model. The following model was used for the randomized complete block design (RCBD) trials:


$$\begin{array}{cc} Y_{ij}\;=\mu +G_{i}+b_{j}+e_{ij} & \{\begin{array}{l} eij\sim N\left(0,\;\sigma^{2}\right)\\ Gi\sim N\left(0,\;\sigma_{G}^{2}\right)\\ bj\sim N\left(0,\;\sigma_{b}^{2}\right) \end{array} \end{array}$$

Where Y_ij_ represents the reflectance data of the wavelength derived from genotype i with block j;

µ represents the overall mean; G_i_ is the random effect of genotype i, b_j_ is the effect of block j, and e_ij_ is the error associated with the observation. All random effects were assumed to have a normal distribution. The following model was used for the alpha lattice trials:


$$\begin{array}{cc} Y_{ijk}\;=\mu +G_{i}+{\text{Rep}}_{j}+\;b_{k(j)}+e_{ijk} & \{\begin{array}{l} eijk\sim N\left(0,\;\sigma^{2}\right)\\ Gi\sim N\left(0,\;\sigma_{G}^{2}\right)\\ bk\sim N\left(0,\;\sigma_{b}^{2}\right)\\ Repj\sim N\left(0,\;\sigma_{b}^{2}\right) \end{array} \end{array}$$

Where Y_ijk_ denotes the reflectance value of each of the wavelengths derived from genotype i in replicate j and block k. Rep_j_ is the effect of the replicate j; b_k(j)_ is the effect of block k nested within replicate j, and e_ijk_ is the error associated with the observation of genotype i in block k within replicate j. All random effects were assumed to have a normal distribution. Variance components estimated above were used to calculate heritability. Broad-sense heritability (*H^2^*) was calculated for root starch content and each wavelength as follows:


$$H^{2}=\frac{\sigma_{g}^{2}}{\sigma_{\text{g}}^{2}+\frac{\sigma_{e}^{2}}{\text{nRep}}}\times 100$$

Where σ_g_^2^ is the genotypic variance; σ_e_^2^ is the residual variance, and nRep is the mean number of repetitions for one genotype in the trial. The estimated heritabilities of the entire measured NIR spectrum were plotted using the ggplot function from the ggplot^2^ package (Wickham, 2016) in R (R Core Team, 2021).

### Instrument comparison

Root spectra were also captured using two additional devices to enable instrument comparison in the five trials from the 2021 harvest season (Trials G, H, I, J, and K) (**Table 1**). These spectrometers include the full range (350 to 2500 nm) portable instrument ASD QualitySpec^®^ Trek (QST; Malvern, Panalytical, Cambridge, UK) with a spectral interval of 1 nm and the benchtop FOSS XDS Rapid Content™ Analyzer NIR spectrometer (BT; FOSS, Hillerød, Denmark) with a spectral range from 400 to 2490 nm and a spectral interval of 10 nm. For the QST, a reference reading was taken when starting a scanning session. Each genotype was measured three times, with each spectrum representing the average of 50 scans. BT spectra were collected in reflectance mode. Three separate samples per genotype were placed in cell quartz glasses and measured three times each. For this spectrometer, each spectrum represents an average of 60 scans. Data from all five trials were combined. Based on the raw spectra from each spectrometer, PCA was used to classify cassava genotypes into homogeneous groups visually. This analysis was performed using the R package factorMineR (Lê et al., 2008). The performance of the three devices to predict root starch content was carried out using the same sample sets. Three approaches were used: (**1**) the initial full spectral range of the three devices; (**2**) comparison of the three devices in the overlapping regions (740−1070 nm); and (**3**) the spectral data from the QST and BT were trimmed at the beginning (< 600 nm) and the end of the spectra (> 1900 nm) to remove potential noise. The selected range was determined after graphical visualization of the raw spectra. Background noise was evident with QST. The BT spectra were trimmed to the same range as the QST spectra for consistency and ease of comparison.

## Results

### Reference data exploration

**Figure 1:**
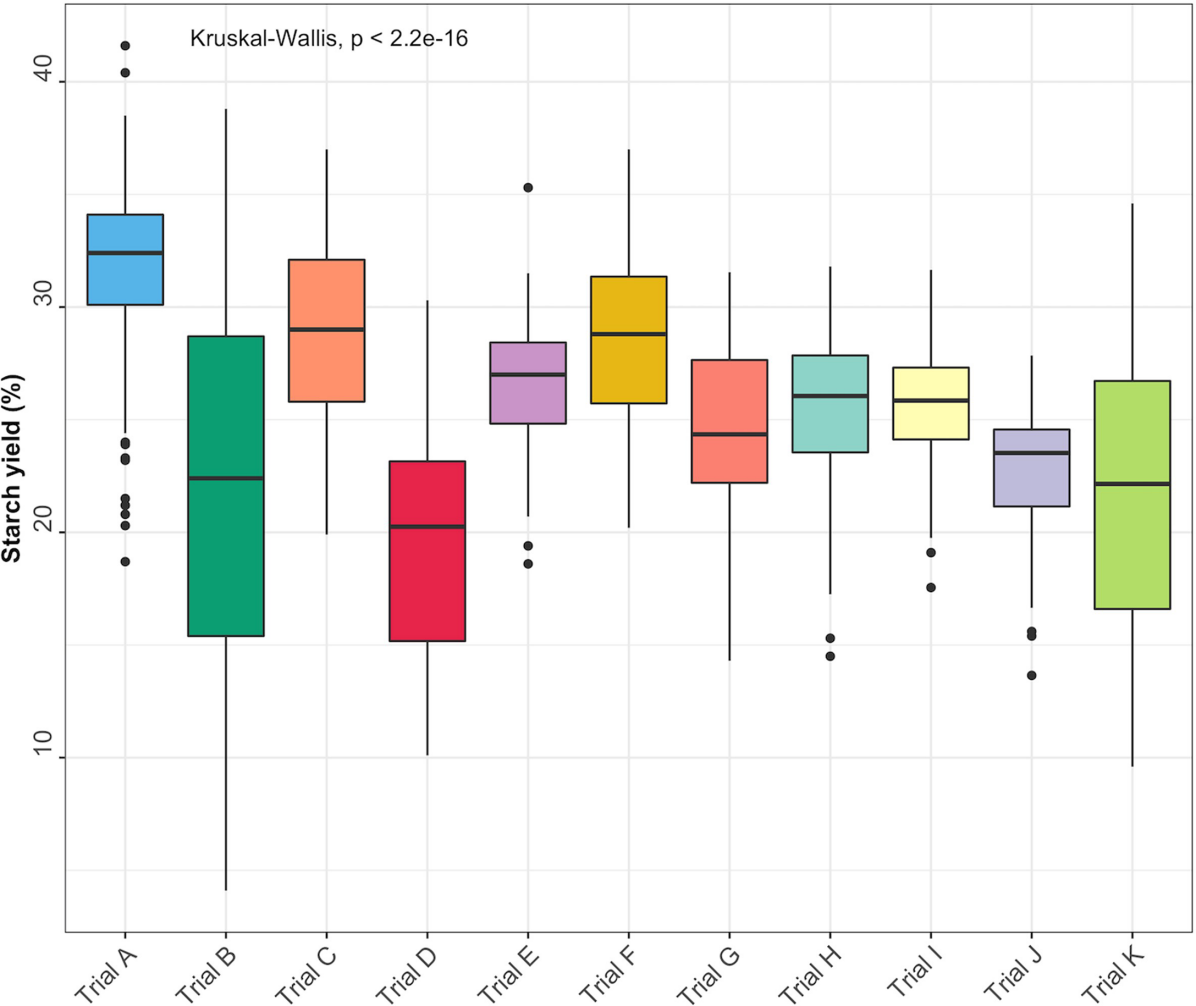
Root starch content distributions for the 11 evaluated cassava trials.

Root starch content ranged from 4.1 to 41.6% among the 344 unique genotypes in this study. Furthermore, we observed root starch content varied within and between trials, over time, and across environments (**Figure 1**). The Kruskal-Wallis rank test revealed significant (*P*< 0.05) differences in starch content between trials. Trial B (coefficient of variation = 0.37) had the most genotype variation, followed by Trial K (coefficient of variation = 0.3). Trial I displayed the lowest level of variability (coefficient of variation = 0.1). **Table 1** shows descriptive statistics for root starch content and the number of genotypes used for calibrating each trial. **Supplementary Table 1** shows the number of common genotypes between trials.

### Principal component analysis of the raw spectral data

**Figure 2:**
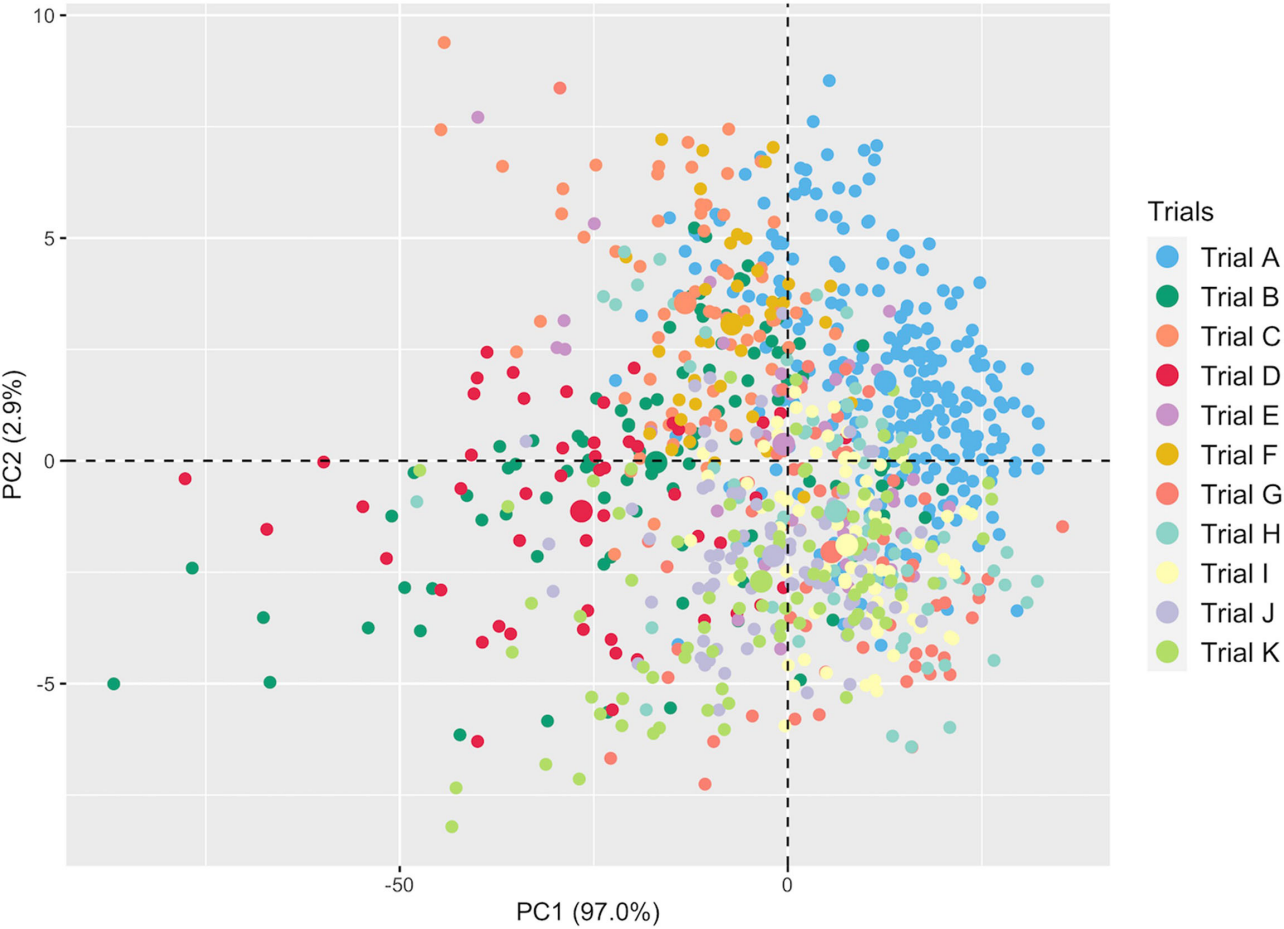
Principal component analysis of NIR spectral data from fresh cassava root scans captured with the SCiO.

A total of 301 averaged scans were recorded using the SCiO device across trials (**Supplementary Table 2**). **Supplementary Figure 1** depicts the averaged raw spectra recorded on ground fresh cassava roots. PCA revealed variation between genotypes and subtle differences between trials (**Figure 2**). The overlap between trials could be attributed to common genotypes present and their close relatedness. The overlap may also be due to overlap in the mean and range of root starch content across trials (**Supplementary Figure 2**). The first PC accounted for 97.0% of the variation in the NIR spectra, while PC2 accounted for 2.9%. Overall, this exploratory PCA revealed the potential of spectral information in characterizing genotypes.

### Analysis of SCiO spectra data using partial least squares regression

#### Assessment of prediction accuracy between trials

**Figure 3:**
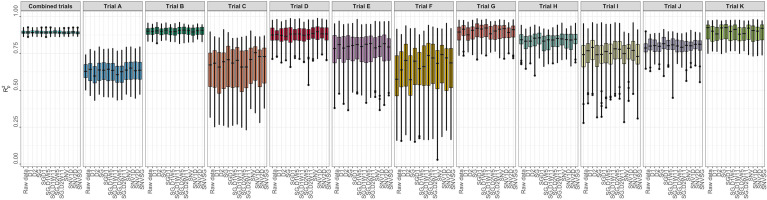
Individual trial performance using partial least squares regression. Pearson’s correlation between predicted and observed values in the test set (R^2^_p_); no spectral pretreatment (raw data); standard normal variate (SNV); standard normal variate and first derivative (SNV1D); standard normal variate and second derivative (SNV2D); first derivative (D1); second derivative (D2); Savitzky-Golay with window size = 11 (SG); standard normal variate and Savitzky-Golay (SNVSG), gap segment derivative with window size = 11 (SGD1), Savitzky-Golay with window size = 5 and first derivative (SG.D1W5); Savitzky-Golay with window size = 11 and first derivative (SG.D1W11); Savitzky-Golay with window size = 5 and second derivative (SG.D2W5); and Savitzky-Golay with window size = 11 and second derivative (SG.D2W11).

Several metrics were used to evaluate model prediction, including *R^2^*_P_, RPD, RPIQ, RMSE, and SEP. These metrics, which indicate model fitness for each trial, are reported in **Supplementary Table 3**. The prediction of root starch content differed between trials (**Figure 3**). A high-quality model should have higher *R^2^*_P_ and *R^2^*_CV_ values, lower RMSE_P_ and RMSE_CV_, and SEP and bias close to zero. Standard guidelines for the interpretation of *R^2^*_P_ (Williams and Norris, 2001; Lebot et al., 2009; Polinar et al., 2019) and RPD (Williams and Norris, 2001; Williams, 2014; Polinar et al., 2019) were applied. Based on the *R^2^*_P_ values for each pretreatment and trial and the *R^2^* interpretation guidelines suggested by Williams and Norris (2001), the trained models could be used for: (a) rough screening (Trial A; *R^2^*_P_ =0.61.-0.64); (b) screening and other approximate calibration (Trials E, I, J; *R^2^*_p_ = 0.71-0.81); (c) most applications but with caution (Trials B, D, G, K, and Combined; *R^2^*_p_ = 0.86-0.90); (d) rough screening to screening and other approximate calibration (Trials C and F, *R^2^*_p_ = 0.59-0.68); and (e) screening and other approximate calibration to use for most applications but with caution (Trial H; *R^2^*_p_ =0.82-0.85). Based on the RPD values, the predicted models could be used for screening (Trials B, K, and Combined; RPD ≥ 3) and very rough screening (Trials D and H; RPD = 1.593 – 2.306) in some trials, but not in others (Trials A, C, E, F, J; RPD = 1.595 - 2.306). A combination of factors, including the small sample range between the reference data and the number of samples used, could have hampered efficient model prediction in trials E and F. Trials E and F are both NCRP trials; the final testing stages before varieties can be commercialized. These trials include superior genotypes with high yield and starch content and little variation because they are all high performers. Variation in the environment is also an important factor that could have influenced model prediction. Trials E and F, which had similar genotypes, were tested at two different phenotyping sites (Ikenne and Ago-Owu). Similar findings apply to Trials C and D, which were also evaluated in two different agroecological zones (Ikenne and Ibadan). Trials B, K, and the combined trials showed good predictive ability (RPIQ ≥ 4.0) consistent with their RPD values (Williams, 2014). The similarity of the RMSE_P_ and RMSE_CV_ for most trials confirmed the fair and robust fitting of the validation samples. Overall, Trials B, D, G, K, and Combined best predicted root starch content. An effect of spectral pretreatment on model prediction was observed in some cases. (**Figure 3**). The spectral pretreatments with the best performing models from Trial B were D2, SGD2W5, and SGD2W11. The model based on SGD1 and SGD1W11 pretreated spectra performed best in Trial G. The optimal model from Trial D was pretreated with SNV1D. The best performing pretreatment in the Combined trial was SG. The variability of genotypes within each trial had a greater impact on model prediction performance (r_s_ = 0.78, *P*< 0.05) than the number of genotypes (r_s_ = 0.18, *P*< 0.05) (**Supplementary Figures 3**, **4**). In terms of prediction, trials with the highest coefficient of variation performed better. Trial A had the most genotypes (261) but a lower *R^2^*_P_ value than Trials B (97), K (88), and D (52), which had smaller sample sizes but high coefficients of variation. As a result, model prediction can be linked to the level of sample variability.

#### Comparison of different prediction models using aggregated data

**Figure 4:**
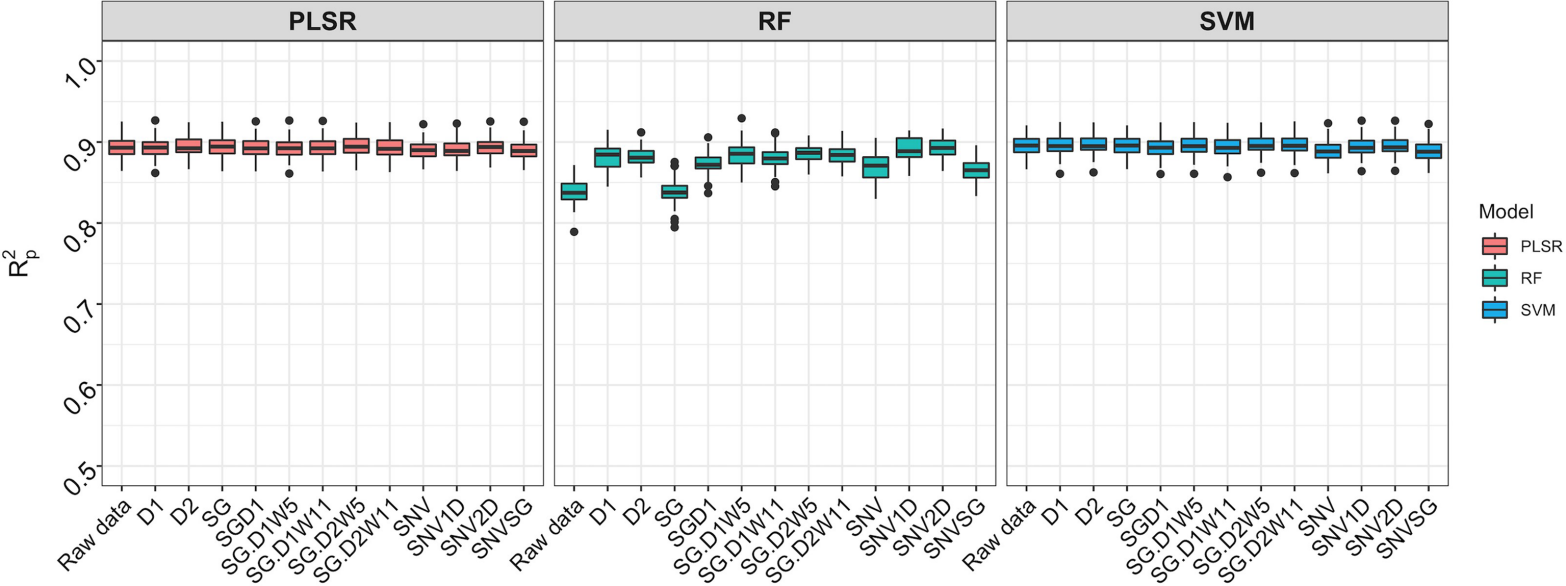
Comparison of three chemometric modeling approaches using SCiO spectral data and all accessions combined. Pearson’s correlation between predicted and observed values in the test set is represented by the y-axis (R^2^_p_). The various pretreatment approaches and the model without spectral pretreatment (raw data) are depicted on the x-axis (standard normal variate (SNV), standard normal variate and first derivative (SNV1D), standard normal variate and second derivative (SNV2D), first derivative (D1), second derivative (D2), Savitzky-Golay with window size = 11 (SG), standard normal variate and Savitzky-Golay (SNVSG), gap segment derivative with window size = 11 (SGD1), Savitzky-Golay with window size = 5 and first derivative (SG.D1W5), Savitzky-Golay with window size = 11 and first derivative (SG.D1W11), Savitzky-Golay with window size = 5 and second derivative (SG.D2W5), and Savitzky-Golay with window size = 11 and second derivative (SG.D2W11)). Three modeling approaches were evaluated: random forest (RF), partial least square regression (PLSR), and support vector machine (SVM) (SVM). SVM and PLSR both produced consistent and comparable predictions.

**Figure 5:**
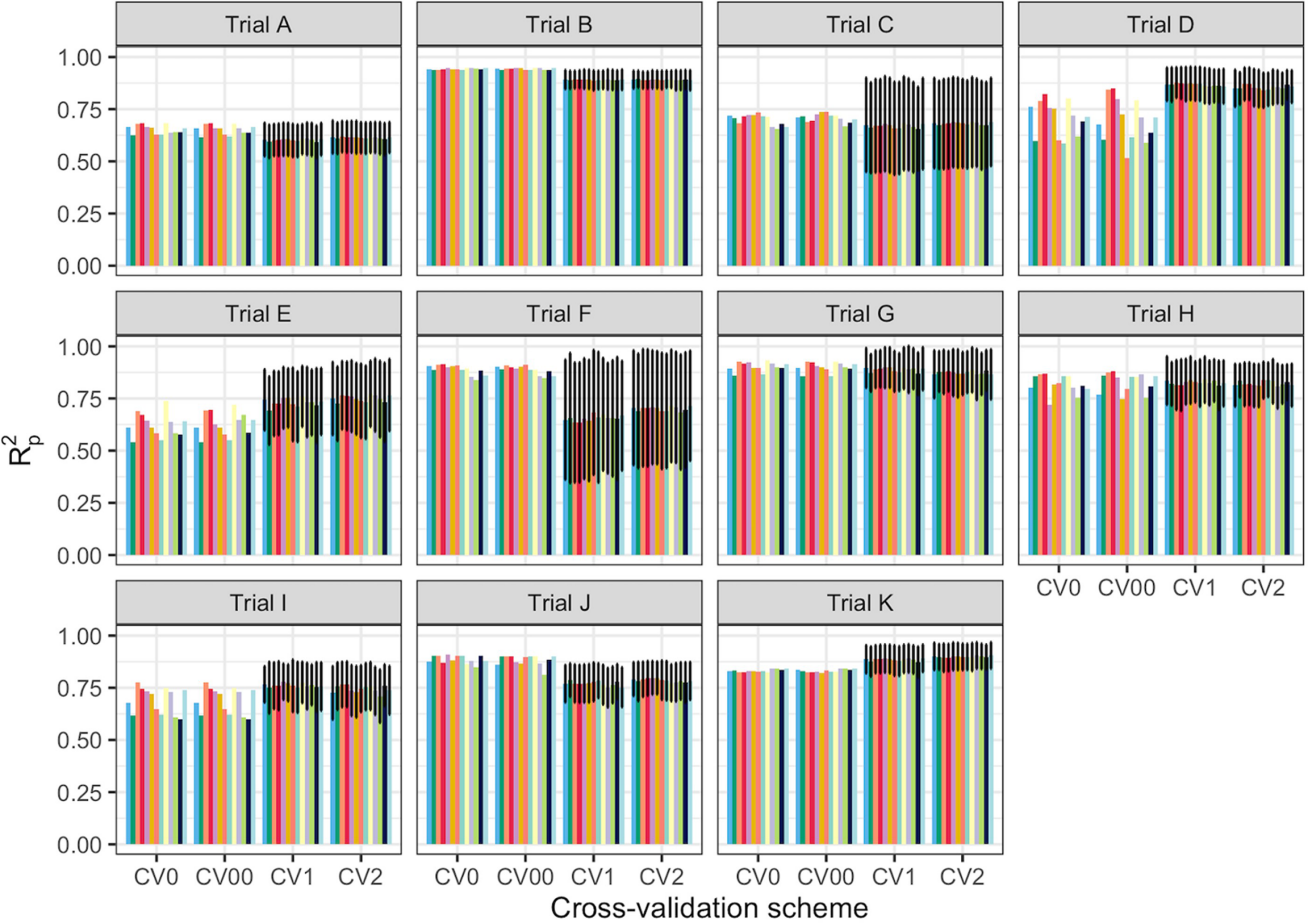
Prediction of cassava root starch content using four cross-validation schemes and the partial least squares regression algorithm. The x-axis displays the four cross-validation (CV) schemes. The y-axis shows the squared Pearson’s correlation between predicted and observed values in the test set (R^2^_p_) for 50 iterations of the waves prediction pipeline with no spectral pretreatment. The colors represent the various pretreatments. CV0 indicates leave-one-trial-out CV and CV00 indicates that there was no overlap between genotypes and environments in the training and test sets. CV1 indicates overlap in the environment but not genotypes between the training and test sets. CV2 indicates an overlap of both genotypes and environments in the training and test sets. However, genotypes with multiple replicates within a trial were sorted together in all cases. Error bars show the standard deviation for schemes with subsampling (CV1 and CV2). As no subsampling occurred in either the CV0 or CV00 schemes, the standard deviation was not calculated and, hence, no error bars are displayed.

Spectral data from all 11 trials were combined and the model prediction was assessed using three chemometric modeling approaches: RF, PLSR, and SVM. Using PLSR, high prediction accuracies were obtained regardless of the pretreatment applied (*R^2^*_P_ = 0.89, RPD > 3.0, RPIQ > 3.9, SEP ≤ 2.07%) (**Figure 4** **;** **Supplementary Table 4**). SVM performance was also consistently satisfactory across the 11 pretreatments. This was supported by a high RPD value (> 3), RPIQ (>3.9), low SEP (≤ 2.07%), and low bias (0.01−0.06) (**Figure 4** **;** **Supplementary Table 4**). Regarding statistical performance parameters, SVM and PLSR models yielded comparable predictions. When the RF model was applied, models based on SNV1D (*R^2^*_P_ = 0.89, RPD = 3.03, RPIQ = 3.97, SEP = 2.07%) and SNV2D (*R^2^*_P_ = 0.89, RPD = 3.04, RPIQ = 4.01, SEP = 2.04%) were deemed reasonable for root starch content prediction, while other pretreatments showed only a fair RPD value (2.5 ≤ RPD ≤ 2.9). The lowest predictability for the RF model was obtained when no spectral pretreatment was applied (*R^2^*_P_ = 0.84, RPD = 2.49, RPIQ = 3.24, SEP = 2.52%) (**Figure 4** **;** **Supplementary Table 4** **)**. Four cross-validation schemes (CV1, CV2, CV0, and CV00) were used to evaluate the ability of each model to correctly predict root starch content across a range of realistic scenarios. **Supplementary Tables 5, 6** show the performance statistics of the models developed using PLSR and SVM. The SVM and PLSR models performed nearly identically across all CV schemes. (**Figure 5**; **Supplementary Figure 5**). The overall mean model performance based on *R^2^*_P_ ranged from 0.76 to 0.79, which is lower than within-trial *R^2^*_P_ (0.89 to 0.90). The performance of other metrics was also lower (**Supplementary Table 7**), but the difference in performance between groups was negligible. Model prediction was slightly improved when the tested set of genotypes was represented in the training set. Likewise, the SEP decreased in the schemes in which the test set environment was represented in the training set (CV1, CV2). The genetic similarity of the genotypes may have contributed to the comparable model performance observed across scenarios.

### Wavelengths of importance and heritability

The variable importance analysis identified the relative contribution of wavelengths in predicting root starch content. The most informative wavelengths for both PLSR and RF models were between 815 and 980 nm, corresponding to a) the third overtone of C-H and C-H_2_ stretching related to the presence of carbohydrates and b) the second overtone for O-H bands, the most prominent signal for water (Bantadjan et al., 2020a; Bantadjan et al., 2020b; Farhadi et al., 2020) (**Table 2**; **Supplementary Figure 6**).

**Table 2:** Top wavelengths identified through variable importance analysis for predicting root starch content with partial least squares regression (PLSR) and random forest (RF) models using data captured with SCiO™ for all combined cassava breeding field trials at IITA.

	Wavelength (nm)												
Model	PLSR		878	879	880	911	912	*959*	*960*		*973*	*974*	*975*
	RF	815				913		963	964	965	979	980	

**Figure 6:**
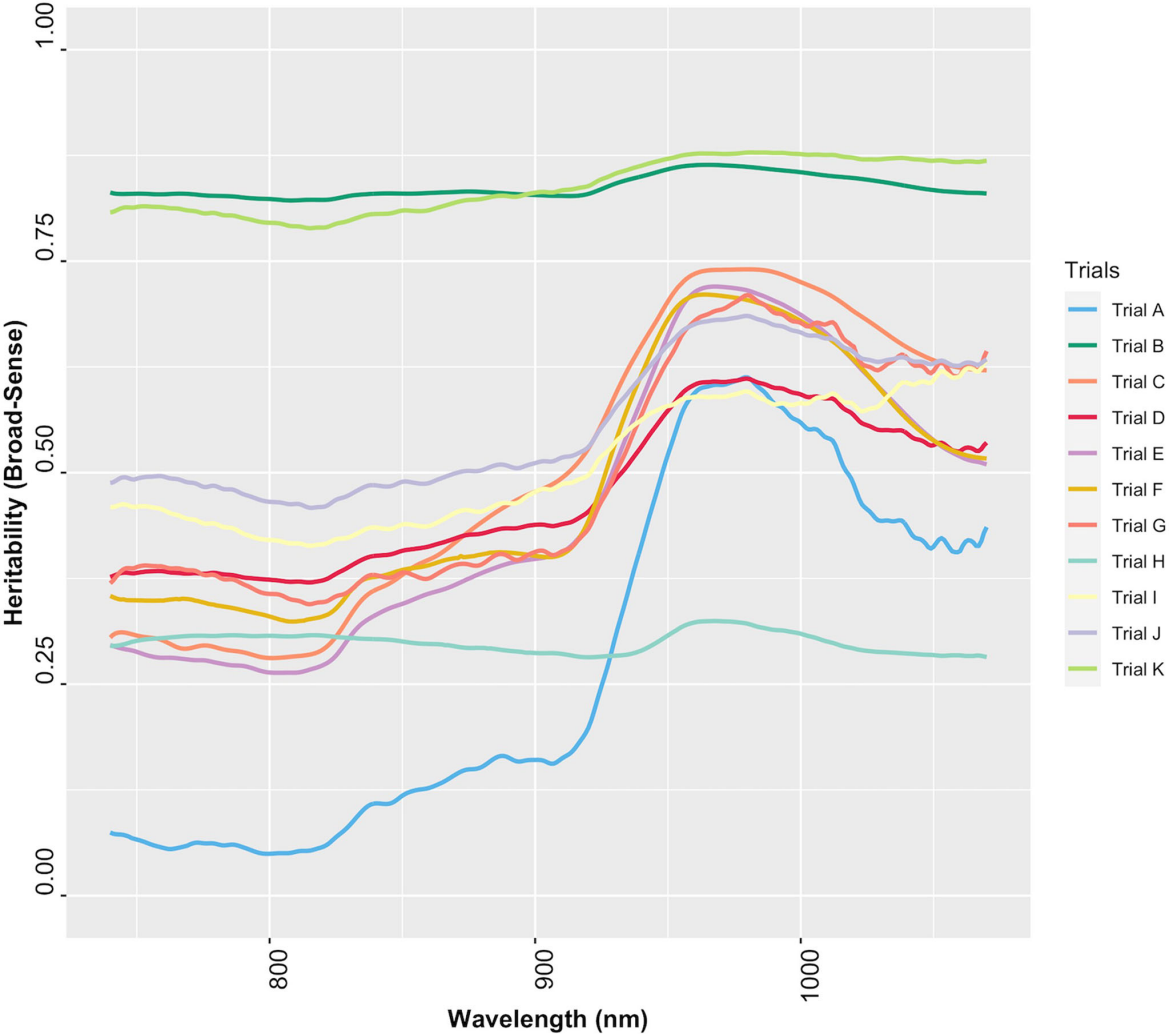
Broad-sense heritability of each wavelength of cassava root NIR spectra collected with the SCiO.

The extent to which NIR spectral variation is due to genetic variation among genotypes was examined by computing the heritability of NIR reflectance values for each trial. The heritability of NIR spectra varied between trials and across spectral regions (**Figure 6**). Trials K and B had higher heritabilities across all wavelengths (*H^2^* ≥ 0.79), whereas Trial H had the lowest range of heritability (*H^2^* < 0.4) (**Supplementary Table 8**). This finding implies that most of the variation in NIR spectral patterns is due to the genetic variation among genotypes. Spectra from 871 to 1070 nm, a range that contains spectral bands strongly related to root starch content (Bantadjan et al., 2020a; Bantadjan et al., 2020b; Farhadi et al., 2020), showed higher heritability (*H^2^* > 0.5) (**Figure 6**). The heritability of root starch content was also computed for each trial. The heritability of root starch content on a mean entry basis ranged from moderate (*H^2^* = 0.53; Trial J) to high (*H^2^* = 0.88; Trial B), except for Trial I, which had a lower heritability (*H^2^* = 0.29). The heritability of root starch content also varied between years and locations (**Supplementary Table 9**). Except for Trial H (*H^2^*: 0.28-0.32), heritability estimates based on spectral data were slightly higher than root starch content heritability estimates, supporting the possibility of using spectral information *via* indirect selection to improve traits in cassava breeding.

### Instrument comparison

**Figure 7:**
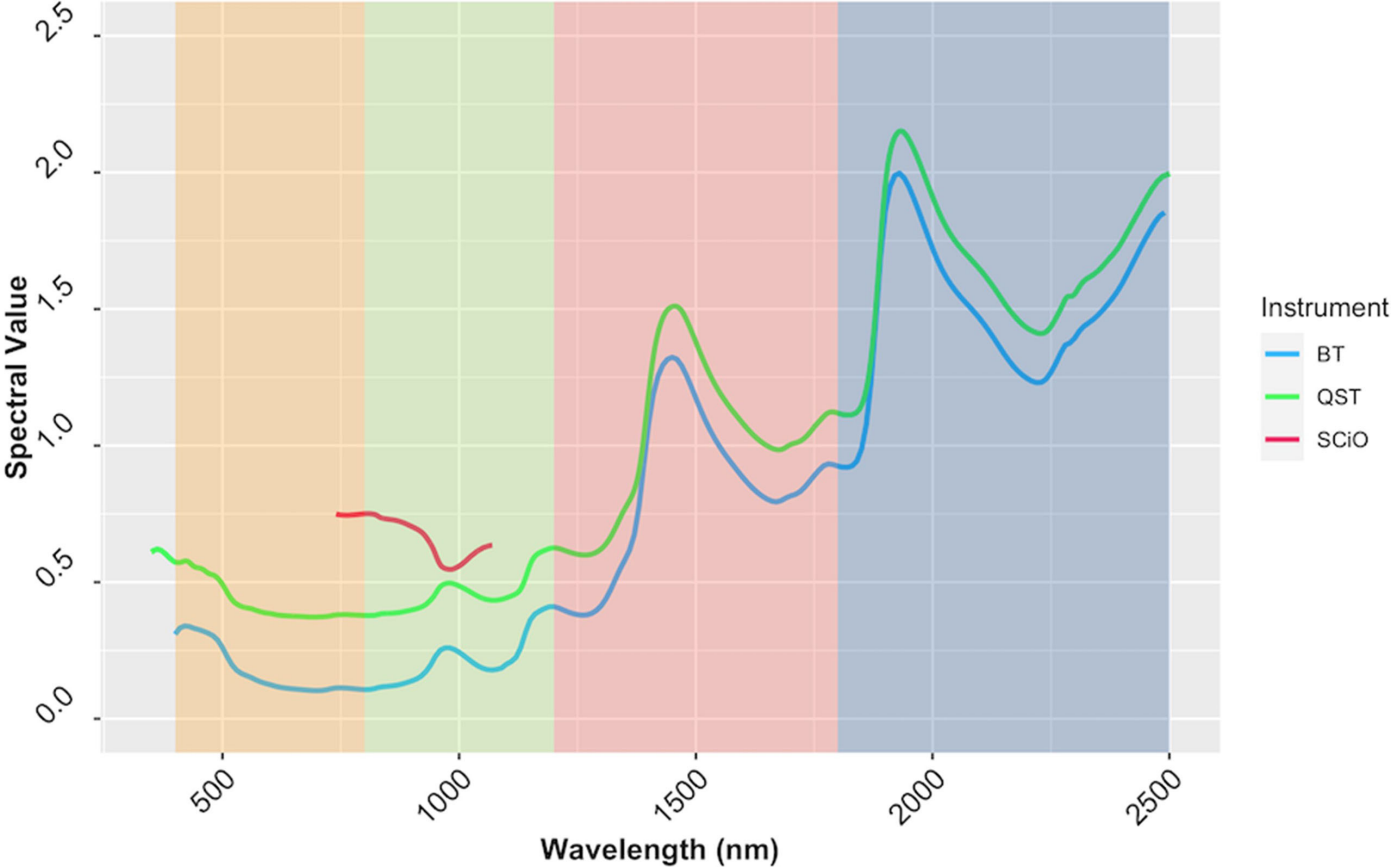
The average spectrum of the cassava accessions obtained using ASD QualitySpec^®^ Trek (QST), Benchtop FOSS XDS Rapid Content™ Analyzer NIR spectrometer (BT) and pocket-sized SCiO™ (SCiO). The various NIRS regions are highlighted on the background, yellow (visible; 400−800 nm), green (region1; 800−1200 nm), pink (region2; 1200−1800 nm), and blue (Region 3; 1800−2500 nm).

Reflectance values varied across wavelengths, but the patterns of reflectance recorded by the QST and BT devices were quite similar. The SCiO patterns, on the other hand, appear to be different, possibly due to distinct optical parameters and operational characteristics of this miniaturized device. Furthermore, the spectral pattern differences could be explained by the proprietary algorithm used to remove noises from the raw signals captured by the SCiO sensor before storing the raw spectral data in the cloud (**Figure 7**). PCA of raw spectra from the different devices revealed further similarities (**Supplementary Figure 7**). For BT, PC1 explained 83.7% of the variability in the raw spectral data, while PC2 explained 9.9%. Similarly, for QST, PC1 accounted for 85% of the variability, while PC2 accounted for 9%. In contrast to BT and QST, PC1 and PC2 captured 97.1 and 2.7% of the variability of the SCiO, respectively. Differences between genotypes and subtle differences between trials were observed regardless of the instrument used (**Supplementary Figure 7**).

When the entire spectral range (SCiO: 740−1070 nm; QST: 350−1070 nm; BT: 400−2490 nm) was used, adequate and consistent prediction was achieved across pretreatments using the SCiO spectrometer with PLSR (*R^2^*_P_ = 0.89-0.90, RPD = 3.10−3.19, RPIQ = 3.74−3.85, SEP = 1.47−1.52%) and SVM (*R^2^*_P_ = 0.89−0.90, RPD = 2.99−3.31, RPIQ = 3.61−4.00, SEP = 1.47−1.52%) models. In general, the RF model statistics were lower (*R^2^*_P_ = 0.77−0.87, RPD = 2.08−2.82, RPIQ = 2.53−3.37, SEP = 1.67−2.22%) (**Supplementary Figure 8**). Model statistics for untrimmed spectral data derived from the BT varied greatly depending on pretreatments and the chemometric model used. The optimal BT PLSR model was obtained when the SGD1W11 pretreatment was applied (*R^2^*_P_ = 0.87, RPD = 2.99, RPIQ = 3.60, SEP = 1.66%), while the highest statistical indicators from RF were obtained when spectra data was processed by SNV2D (*R^2^*_P_ = 0.89, RPD = 3.06, RPIQ = 3.70, SEP = 1.54%) and SNV1D (*R^2^*_P_ = 0.89, RPD = 3.03, RPIQ = 3.64, SEP = 1.56%). The best pretreatment approach for the SVM model was SG (*R^2^*_P_ = 0.89, RPD = 3.12, RPIQ = 3.77, SEP = 1.52%), followed by SGD1 (*R^2^*_P_ = 0.88, RPD = 3.11, RPIQ = 3.75, SEP = 1.56%). Less optimal calibration models were observed with the QST spectrometer (*R^2^*_P_ =0.10-0.83, RPD = 0.99-2.57, RPIQ =1.20-3.10, SEP = 1.89-4.68%) (**Supplementary Table 10**).

**Figure 8:**
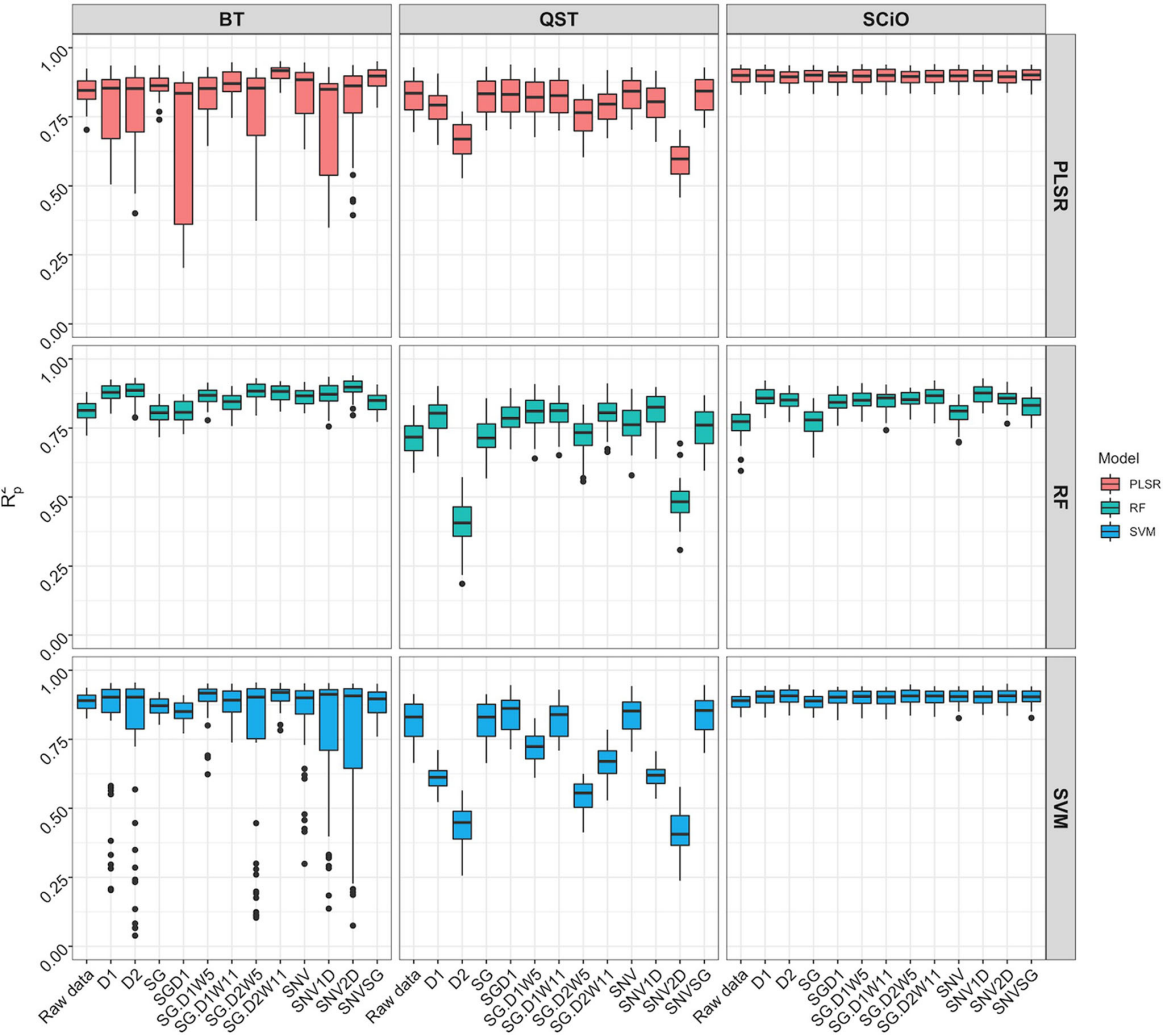
Comparison of model prediction using Partial least squares regression (PLSR), support vector machine (SVM) and random forest (RF) algorithms between ASD QualitySpec^®^ Trek (QST), the Benchtop FOSS XDS Rapid Content™ Analyzer NIRS spectrometer (BT) and the pocket-size SCiO™ (SCiO) using the overlapping wavelengths (740 1070 nm) between the three devices. The Y-axis shows the squared Pearson’s correlation between predicted and observed values in the test set (*R* ^2^_P_). The X-axis indicates the model without spectral pretreatment (raw data) and the different pretreatment approaches used [standard normal variate (SNV), standard normal variate and first derivative (SNV1D), standard normal variate and second derivative (SNV2D), first derivative (D1), second derivative (D2), Savitzky-Golay with window size = 11 (SG), standard normal variate and Savitzky-Golay (SNVSG), gap segment derivative with window size = 11 (SGD1), Savitzky-Golay with window size = 5 and first derivative (SG.D1W5), Savitzky-Golay with window size = 11 and first derivative (SG.D1W11), Savitzky-Golay with window size = 5 and second derivative (SG.D2W5), and Savitzky-Golay with window size = 11 and second derivative (SG.D2W11)].

When the overlapping region between the three devices (740−1070 nm) was used, model prediction with the BT and QST spectrometers improved considerably (**Figure 8**). Model statistics revealed that depending on the pretreatment applied, certain models were suitable for predicting root starch content and, in some cases, were slightly superior to the model developed with the SCiO (**Supplementary Table 11**). The optimal models for the BT were obtained using the SVM (*R^2^*_P_ = 0.91; RPD = 3.39; RPIQ = 4.19, SEP = 1.39) and PLSR (*R^2^*_P_ = 0.91; RPD = 3.37; RPIQ = 4.17, SEP = 1.38) algorithm. Here as well, the QST produced the models with the poorest performance (*R^2^*_P_ = 0.40−0.85; RPD = 1.06−2.70; RPIQ = 1.28−3.26, and SEP = 1.80−4.40%) (**Figure 8** and **Supplementary Table 11**).

After trimming the spectra to remove noise (**Supplementary Figure 9** **)**, model calibration obtained with the BT spectrometer was slightly superior (*R^2^*_P_ = 0.82−0.91; RPD = 2.37−3.52; RPIQ = 2.86−4.35; SEP = 1.32−1.98%) to the model developed with the SCiO (*R^2^*_P_ = 0.77−0.90; RPD = 2.08−3.31; RPIQ = 2.53−4.00; SEP = 1.43−2.22%) depending on the pretreatment and the chemometric model used (**Supplementary Figure 10**, **Supplementary Table 12**). It is critical to use the appropriate pretreatments to achieve more accurate predictions. Regarding root starch content prediction, although the effect of pretreatment on model prediction was more pronounced when using the benchtop device, both the BT and SCiO outperformed QST. Even though the SCiO sensor only captures information in the second and third overtones, its limited spectral range did not affect root starch content prediction in this study. **Supplementary Tables 13–15** show the average prediction value from multiple predictions from a random sampling of the test set, while **Supplementary Figure 11** shows the correlation between the obtained predicted values and the reference values. However, newly selected samples from the next harvesting season would accurately correlate the observed laboratory values with the root starch content obtained from the three devices.

## Discussion

### Trial selection, sample coverage, and model prediction

Breeding programs devoted to developing cassava varieties with high root starch content for industry necessitate robust, fast, and low-cost methods for screening breeding populations, particularly at the early stages of selection when many entries are evaluated. Laboratory-based quantification of root starch content is tedious and time-consuming. The potential of NIRS technology for quantifying root starch content was investigated. The importance of training set composition, including consideration of the trial type and phenotypic variation within a trial, was demonstrated in developing a robust model. The current study found that some trials with more genotypes (e.g., Trial A) had lower prediction accuracy than trials with fewer genotypes and a wider range of root starch content (e.g., Trials B and K), highlighting the importance of capturing a diverse range of phenotypes (Cafferky et al., 2020; Zerihum et al., 2020). Environment factors may also impact trait prediction. This is evidenced by Trials C and D, which were carried out in two distinct agroecological zones. Trial C was conducted in Ikenne (a rainforest) and Trial D was conducted in Ibadan (a derived savanna). The effects of edaphic and climatic conditions on cassava root content and their physiochemical properties have been previously reported (Benesi et al, 2004; Gu et al., 2013). For a robust model, selecting a set of genotypes representative of the breeding pool from different selection stages, locations, growing seasons, and years is preferable to maximize the number of genotypes. Routine model updates capturing new variations are advised to prevent bias (Lebot, 2012).

## Assessment of model prediction

Various pretreatments were used to correct spectral data. A recent study by Hershberger et al. (2022) evaluated the ability of the SCiO to predict cassava root dry matter content. Consistent results were obtained across the same 12 combinations of pretreatments with PLSR and SVM, but the effect of spectral pretreatment was evident with the RF model. Previous research has highlighted the effect of pretreatment on prediction accuracy for NIRS (Agussabti et al., 2020; Cafferky et al., 2020). Because there is no one-size-fits-all pretreatment, care should be taken to avoid model bias when selecting a spectral pretreatment method.

A thorough evaluation is critical to ensure that models are appropriate for their intended uses. *R* ^2^_p_ and *R* ^2^_cv_ are commonly used to assess model fit and predictive strength, but these metrics should not be used as stand-alone indicators of model performance. RPD and RPIQ are additional statistical parameters used in the current study to evaluate model prediction accuracy. RPD is inappropriate when the assumption of a normal distribution is violated, and its interpretation varies from study to study (Lebot, 2012; Williams, 2014; Zerihum et al., 2020; Zhao et al., 2021). As a result, RPIQ was also considered when evaluating model fit (Bellon-Maurel et al., 2010). Other meaningful metrics to measure model fit that were examined include RMSE, which gives the standard residual error, model SEP, bias, and CCC.

Algorithm choice is also critical for model development. We found that SVM models performed similarly to those trained with PLSR, a more traditional NIRS modeling approach. This is consistent with the findings of Mendez et al. (2019) and Hershberger et al. (2022). They reported a marginal improvement in predictive ability for SVM over PLS, contradicting Ludwig et al. (2019) and Wang et al. (2021) who found SVM superior to PLSR. The observed variation in algorithm performance between studies could be attributed to differences in the trait investigated, data distribution, and sample variability (Frizzarin et al., 2021). Consistent with previous studies, SVM and PLSR outperformed the RF algorithm in this study (Mendez et al., 2019; Abincha et al., 2021; Hershberger et al., 2022). PLSR may remain the go-to model for trait prediction with NIRS due to its sensitivity and computational efficiency.

A decrease in model prediction accuracy was observed when tested with additional cross-validation schemes. While it is important to adequately account for environmental and genotype variability to ensure broad-based calibration, it is equally important to minimize sample bias through an adequate calibration set and genotype representativeness (Au et al., 2020; Hershberger et al., 2022). Within-trial cross-validation should be interpreted cautiously because it can produce overly optimistic statistics and may not reflect the conditions observed in practice. Thus, the four additional cross-validation schemes tested may provide a more realistic assessment of the ability of the SCiO to predict unknown samples (Li et al., 2018; Patel et al., 2020).

## The wavelength of importance and heritability

Important wavelengths for predicting cassava root starch content were identified between 815 and 980 nm through variable importance analysis. This interval has previously been linked to spectral bands associated with starch and water absorption (Bantadjan et al., 2020a; Wang et al., 2021). In this interval, the third overtone associated with C-H, C-H_2_ stretching was reported at 900, 910, 914, 915, and 930 nm (Bantadjan et al., 2020a). A signal from water O-H bonds was captured between 970 and 975 nm (Bantadjan et al., 2020a; Bantadjan et al., 2020b; Farhadi et al., 2020). The peak at 980 nm is likely related to carbohydrates and water in the root samples (Wang et al., 2021). Given that variable importance is used to identify wavelengths that may correspond to the most relevant information for predicting phenotypes, the preferential targeting of these identified wavelengths of importance could simplify the modeling process. Fitting fewer wavelengths would also require less computing time (Li et al., 2020; Wang et al., 2021).

Although broad-sense heritability estimates varied across the NIR spectrum, highly heritable regions were identified. This indicates that NIR spectral bands are influenced mainly by genetic effects (Hein and Chaix, 2014). Heritable NIR signatures, especially those also predictive of root starch content, could be used to identify desirable cassava genotypes (Hein and Chaix, 2014). Highly heritable spectral regions may also aid in deciphering root starch content genetics (Fujimoto et al., 2015; Razar et al., 2021). Such findings highlight the utility of spectral data in conjunction with, for example, genomics-assisted breeding approaches.

## Instrument comparison

Miniaturized NIR spectrometers have the potential to offer more cost-effective and appropriate high-throughput phenotyping procedures for plant breeding programs. Their effectiveness, however, is still under debate. Despite its limited spectral range, more accurate predictions were obtained using the pocket-sized SCiO compared to the QST, regardless of the pretreatment method applied. This contradicts the hypothesis that spectrometers with broader spectral ranges can provide superior predictions. When models trained with the overlapping region of the three devices (740−1070 nm) were compared, the SCiO still had an advantage, as evidenced by its higher predictive ability. The overlapping region may contain the most influential bands for predicting root starch content. Bittante et al. (2021) made a similar observation, emphasizing the importance of capturing the most informative portion of the spectrum. Rukundo et al. (2021) reported that the limited spectral range of the smartphone NIR spectrometer used in their study did not affect model performance. The improved prediction obtained after spectral trimming could be attributed to an increase in signal-to-noise ratio, emphasizing the negative effect of the discarded spectral regions. The poor performance of the QST in all scenarios could be attributed to complex information captured, making their extraction more difficult. Differences in device technology and operational characteristics cannot be ruled out as potential contributors to model prediction disparities between instruments (Stocco et al., 2019; Ozaki et al., 2020; Beć et al., 2021). The number of reports in the literature on using a miniaturized SCiO sensor for trait prediction is growing. Some studies have pointed out the strong performance of spectral data from the SCiO in trait prediction (Li et al., 2018; Riu et al., 2020; McVey et al., 2021; Hershberger et al., 2022), while others have found models developed using SCiO data to be unreliable (Berzaghi et al., 2021). In other cases, the analytical performance of the SCiO sensor was comparable to that of widely used benchtop devices, the go-to instruments in NIR spectroscopy (Li et al., 2018; Wiedemair et al., 2019).

## The routine use of near-infrared spectroscopy for trait prediction in cassava breeding

Recent studies have reported the value of NIRS for predicting key cassava traits such as dry matter, carotenoids, cyanogenic glucosides, and starch content in fresh cassava roots (Sánchez et al., 2014; Ikeogu et al., 2019; Bantadjan et al., 2020a; Bantadjan et al., 2020b; Abincha et al., 2021; Hershberger et al., 2022). NIR sensors, particularly miniaturized devices, will be helpful in cassava breeding programs where thousands of samples are processed, and data turnaround is critical. A significant amount of time spent on starch extraction will be saved. Another anticipated benefit of routinely implementing NIRS technology in cassava breeding is lower selection costs and a lower risk of advancing lines with inadequate starch content. Aside from analytical performance, the cost of technology is an essential factor that influences its adoption and use. The SCiO sensor is much cheaper than the QST and the BT and could appeal to breeding programs with limited resources. However, one potential barrier to the routine use of this device by programs with limited budgets is access to cloud-based data storage. This necessitates a license and the need to operate *via* an internet connection, which is impractical in remote breeding sites. One crucial point to emphasize is that it is misleading to believe that the ability of SCiO to predict traits such as dry matter content and starch content implies that it applies to all traits. The situation may be different for other traits. As a result, the device’s ability to predict other traits should be assessed on a case-by-case basis.

## Conclusion

The ability of the pocket-size SCiO™ spectrometer to predict starch content was investigated, and its performance compared to that of the Benchtop FOSS XDS Rapid Content ™ Analyzer and ASD QualitySpec Trek^®^. The relevance of spectral information was also evaluated. The SCiO sensor successfully predicted starch content in fresh, shredded cassava roots despite its limited spectral range. After removing noise at the beginning and end of the spectrum, model calibration using the BT spectrometer slightly outperformed the SCiO sensor. With the QST, suboptimal calibration was achieved. The SCiO could be an economically viable solution for breeding programs with limited resources looking for a quick analytical tool to predict cassava root starch content. We demonstrated that spectral information could also characterize accessions. The heritability of the spectra highlighted the possibility of using spectral information for quantitative genetic analyses and improvement. Capturing new variations and continual prediction model updates will help ensure adequate predictive performance and avoid incorrect decisions caused by a miscalibrated model.

## Data availability statement

The raw data supporting the conclusions of this article will be made available by the authors, without undue reservation.

## Author contributions

EM designed the study, analyzed the data, wrote the first manuscript, reviewed, and edited it. JH provided critical assistance in data analysis, as well as critically reviewed and edited the manuscript. PP coordinated the experiment and curated the data. AI, KO, and KN collected spectra data. RA collected root starch content data. EA and MA participated in designing some of the experiments and provided critical edits. The manuscript was reviewed and edited by AA, SK, BM-D, EP, PK, and CE. MG provided software assistance and critical edits. IR conceptualized, and coordinated the experiments, as well as edited the manuscript. All authors contributed to the article and approved the submitted version.

## Funding

The authors thank the UK’s Foreign, Commonwealth & Development Office (FCDO) and the Bill and Melinda Gates Foundation (Grant INV-007637, http://www.gatesfoundation.org) for their financial support. This work has been supported by USDA NIFA AFRI EWD Predoctoral Fellowship 2019-67011-29606 (J.H.) and NSF BREAD IOS-1543958 (M.A.G.). This study was also made possible by the support of the American People provided to the Feed the Future Innovation Lab for Crop Improvement through the United States Agency for International Development (USAID). The contents are the sole responsibility of the authors and do not necessarily reflect the views of USAID or the United States Government. Program activities are funded by USAID under Cooperative Agreement No. 7200AA-19LE-00005 (M.A.G.).

## Acknowledgments

We acknowledge the IITA Nigeria Cassava Breeding team at Yam Barn and the Food Science team for the logistic and support provided during harvesting and data collection; the RTBfoods project (https://rtbfoods.cirad.fr):” Breeding RTB products for end-user preferences (RTBfoods)” led by the French Agricultural Research Centre for International Development (CIRAD), Montpellier, France, specifically Dominique Dufour for initial discussions that led to the initiation of this research.

## Conflict of interest

The authors declare that the research was conducted in the absence of any commercial or financial relationships that could be construed as a potential conflict of interest.

## Publisher’s note

All claims expressed in this article are solely those of the authors and do not necessarily represent those of their affiliated organizations, or those of the publisher, the editors and the reviewers. Any product that may be evaluated in this article, or claim that may be made by its manufacturer, is not guaranteed or endorsed by the publisher.

## Footnotes

1
https://www.businesswire.com/news/home/20220408005379/en/Global-Industrial-Starches-Market-2022-to-2027—Growth-Trends-COVID-19-Impact-and-Forecasts—ResearchAndMarkets.com
