# Supplementary material for: Predicting starch content in cassava fresh roots using near-infrared spectroscopy
Source: Front Plant Sci. 2022 Nov 8;13:990250. doi: 10.3389/fpls.2022.990250 (PMC9679500; doi:10.3389/fpls.2022.990250)
Supplement: Supplementary file 2 [file Table_1.docx]

Supplementary References:

Endelman, J. B. (2011). Ridge Regression and Other Kernels for Genomic Selection with R Package rrBLUP. Plant Genome 4, 250-255. doi: 10.3835/plantgenome2011.08.0024. doi:10.3835
