# Supplementary figures and images for: Predicting starch content in cassava fresh roots using near-infrared spectroscopy
Source: Front Plant Sci. 2022 Nov 8;13:990250. doi: 10.3389/fpls.2022.990250 (PMC9679500; doi:10.3389/fpls.2022.990250)

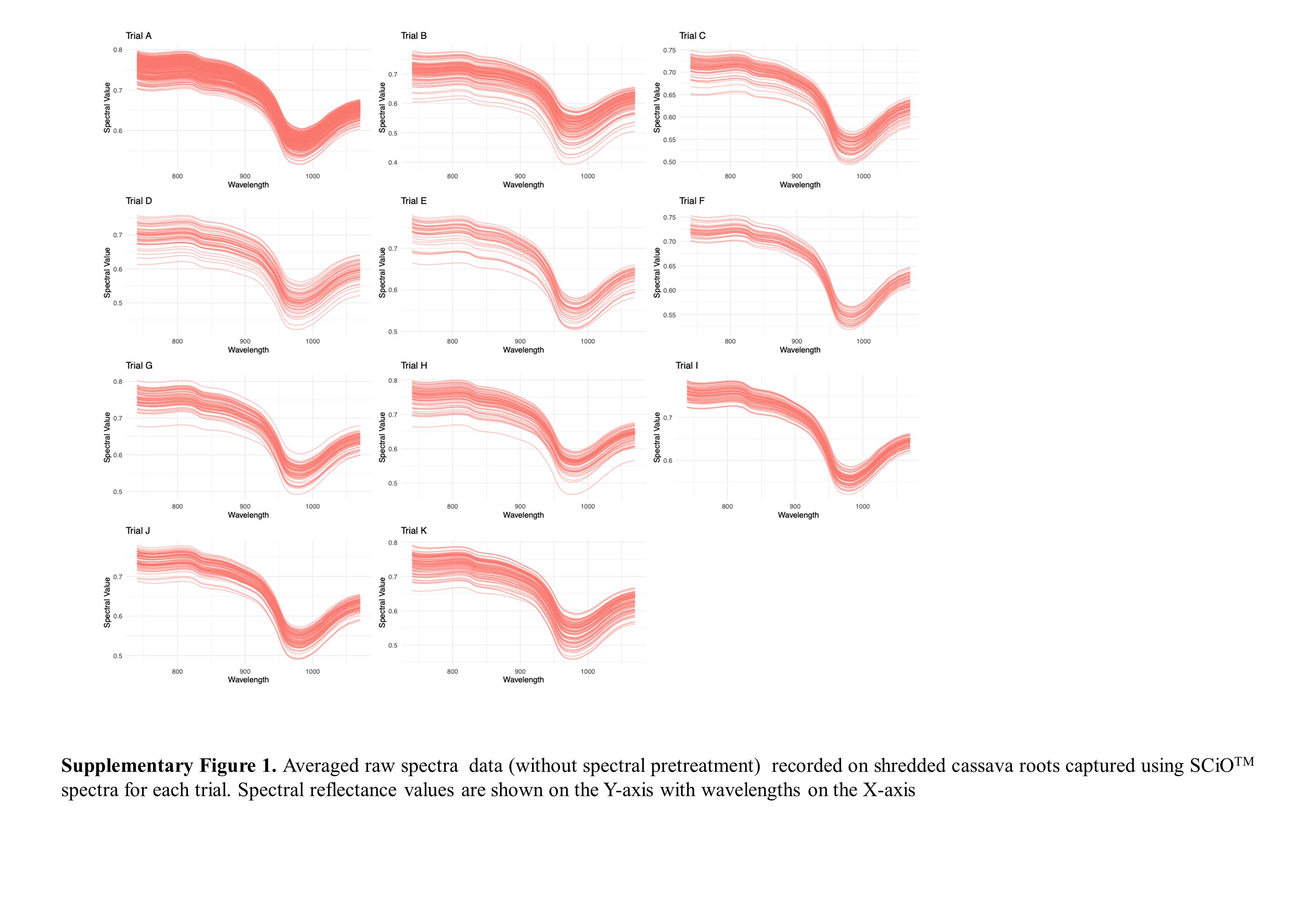

Supplement: Supplementary file 3 [file Image_1.jpeg]

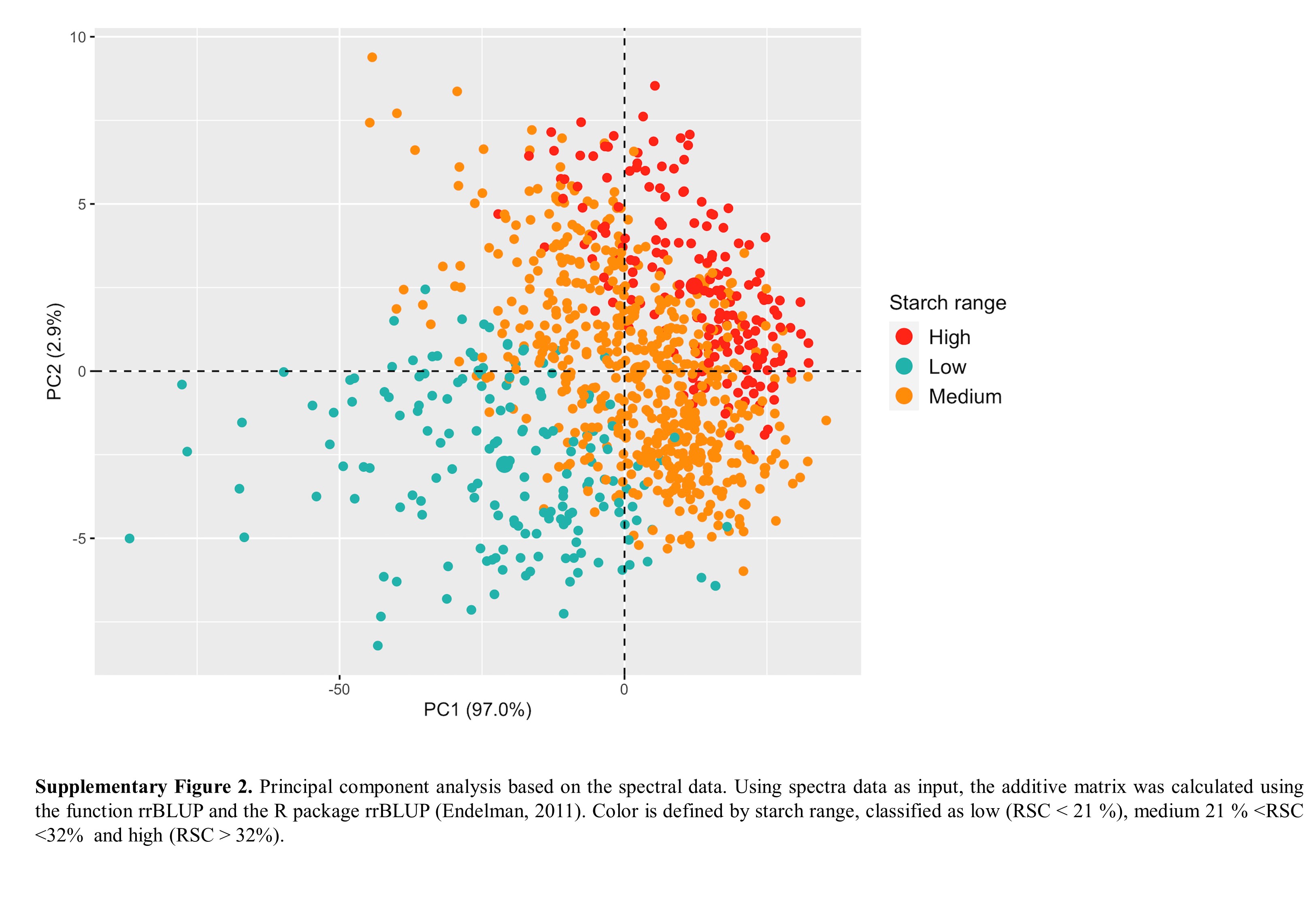

Supplement: Supplementary file 4 [file Image_2.jpeg]

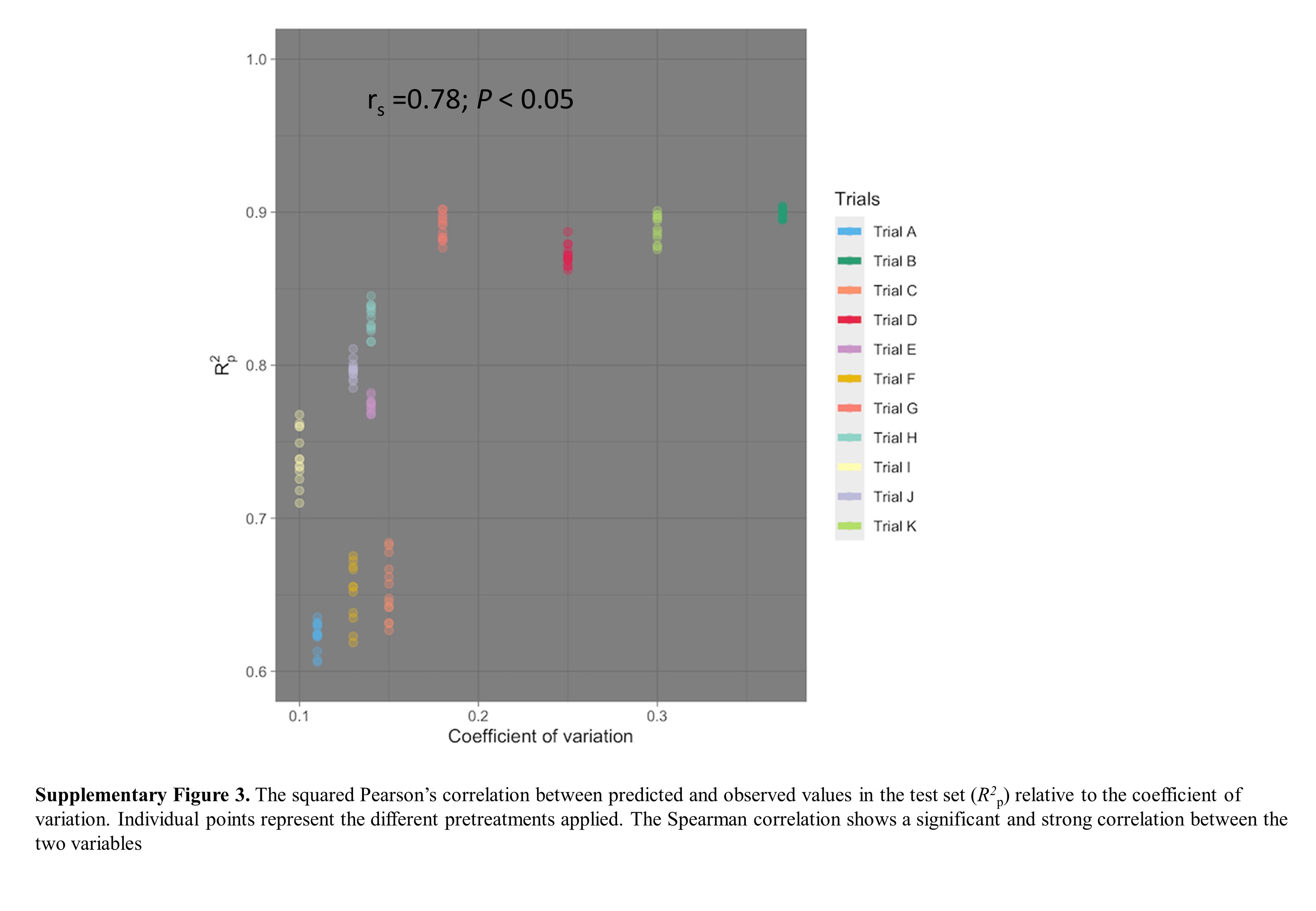

Supplement: Supplementary file 5 [file Image_3.jpeg]

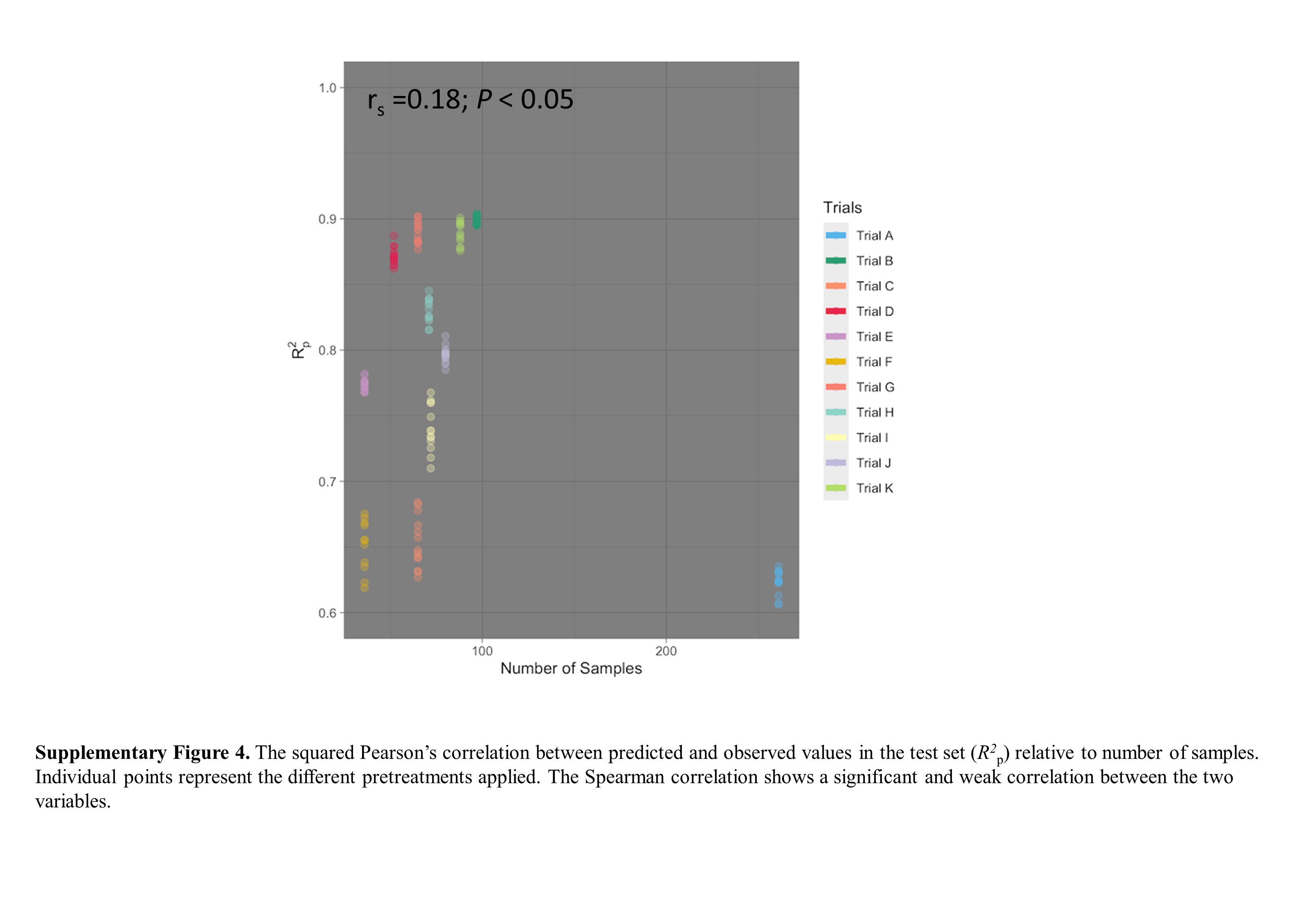

Supplement: Supplementary file 6 [file Image_4.jpeg]

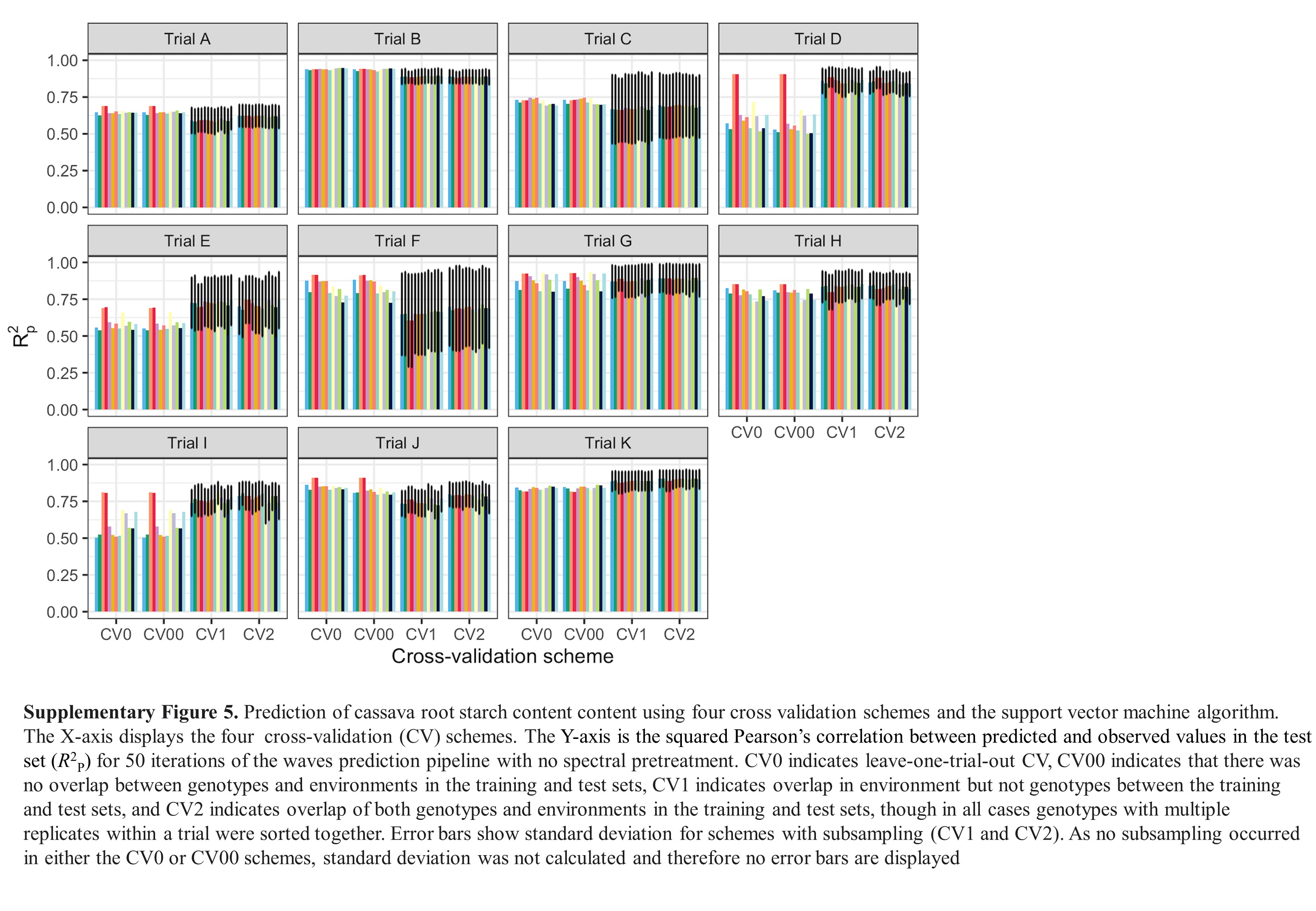

Supplement: Supplementary file 7 [file Image_5.jpeg]

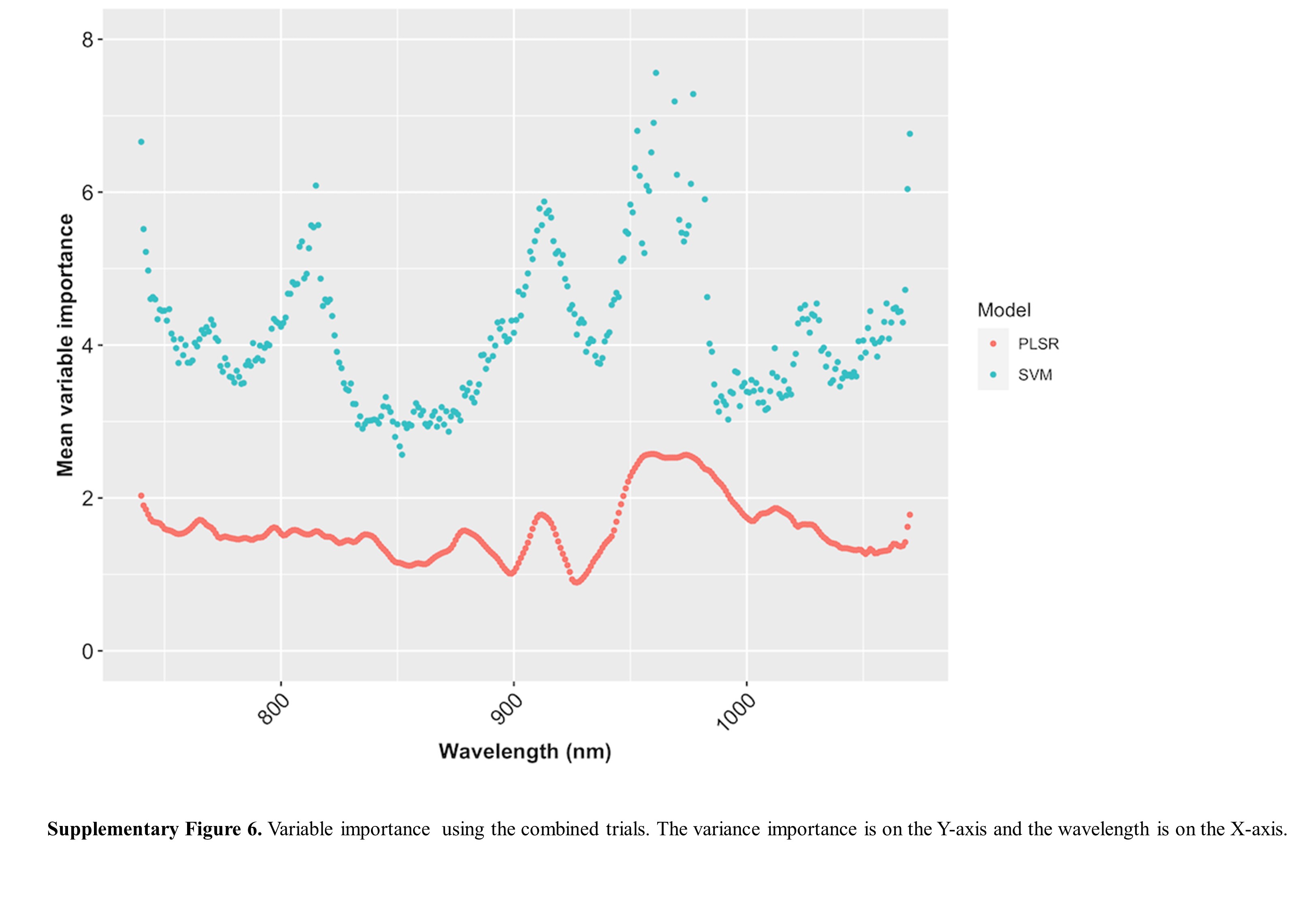

Supplement: Supplementary file 8 [file Image_6.jpeg]

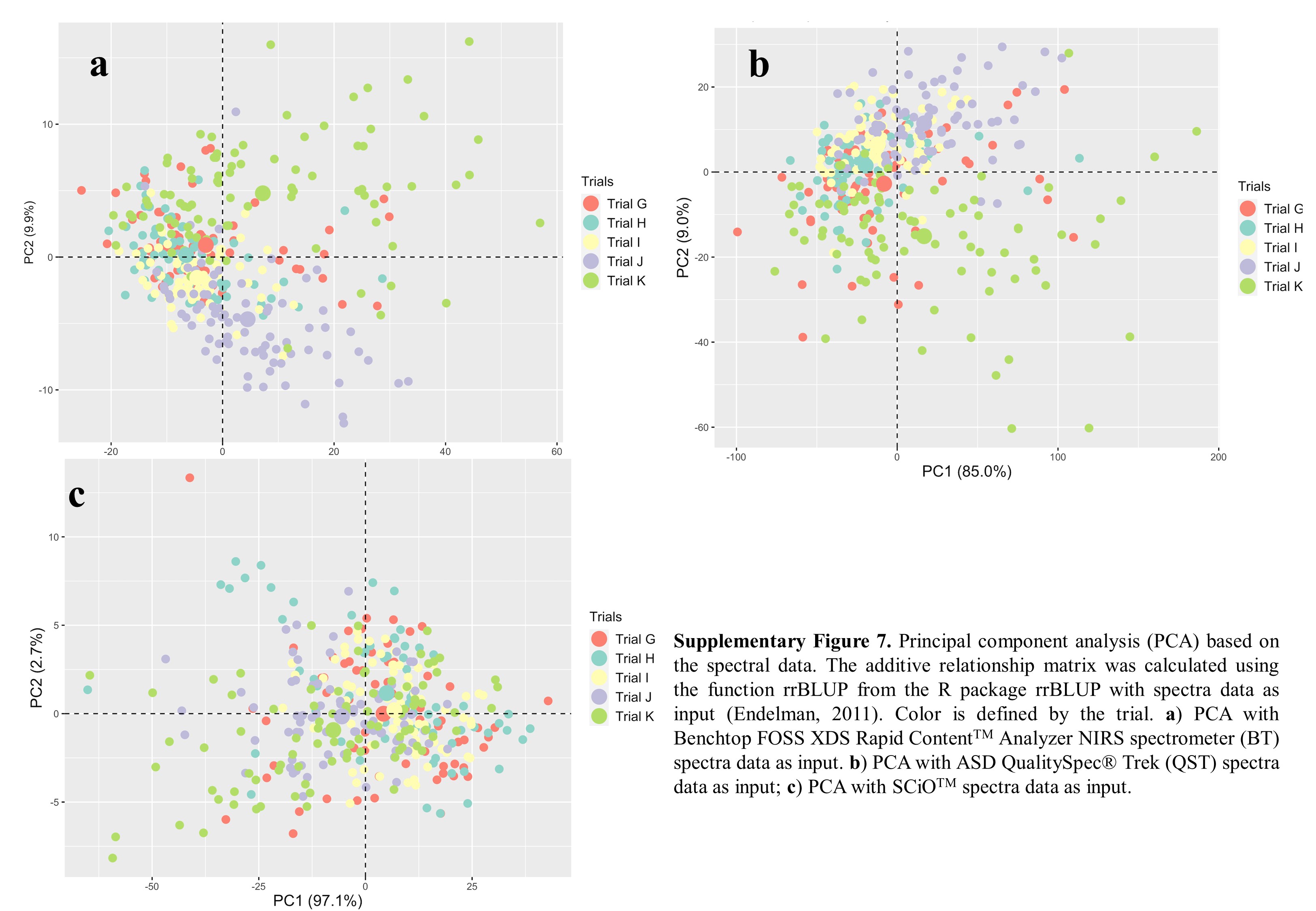

Supplement: Supplementary file 9 [file Image_7.jpeg]

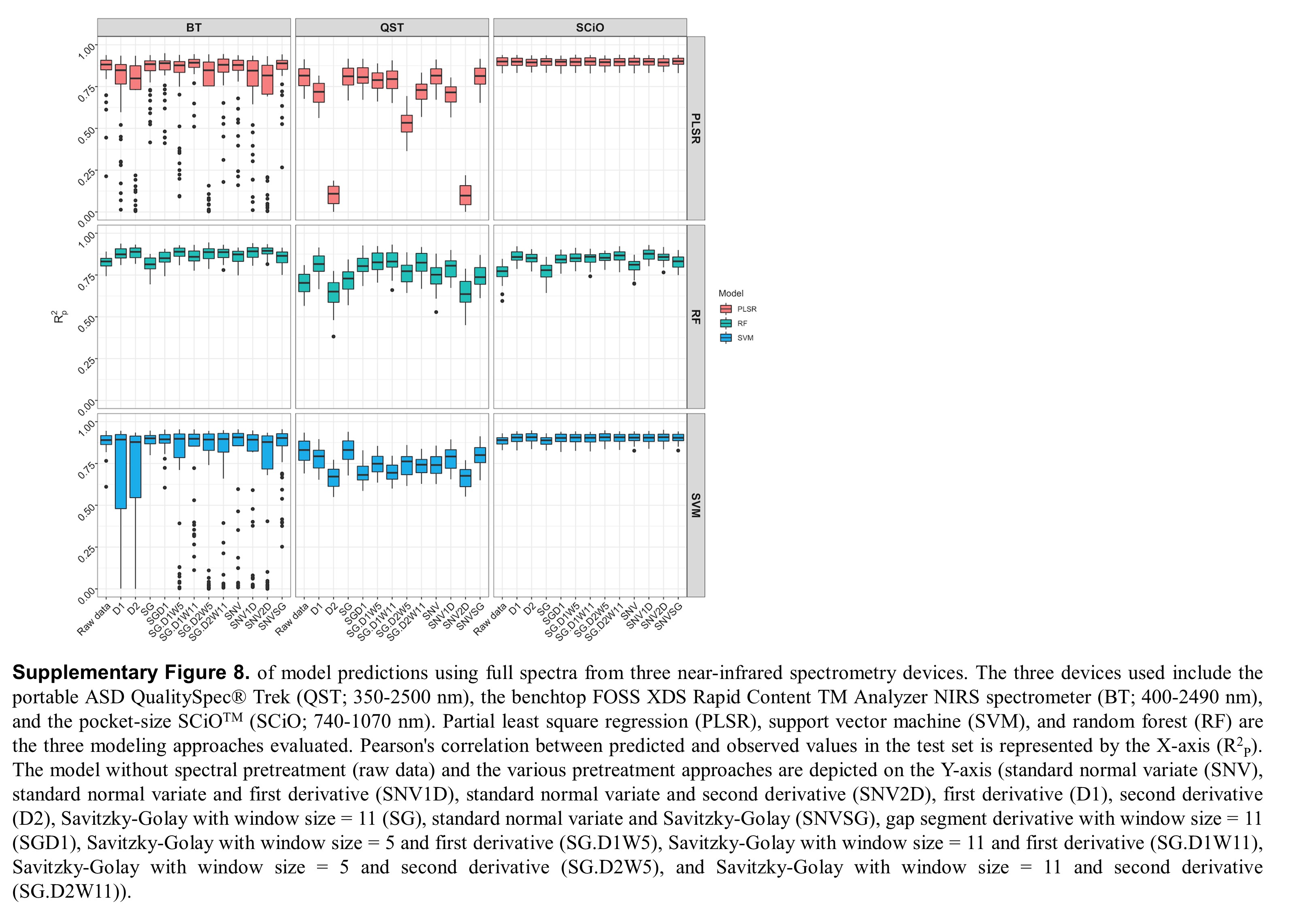

Supplement: Supplementary file 10 [file Image_8.jpeg]

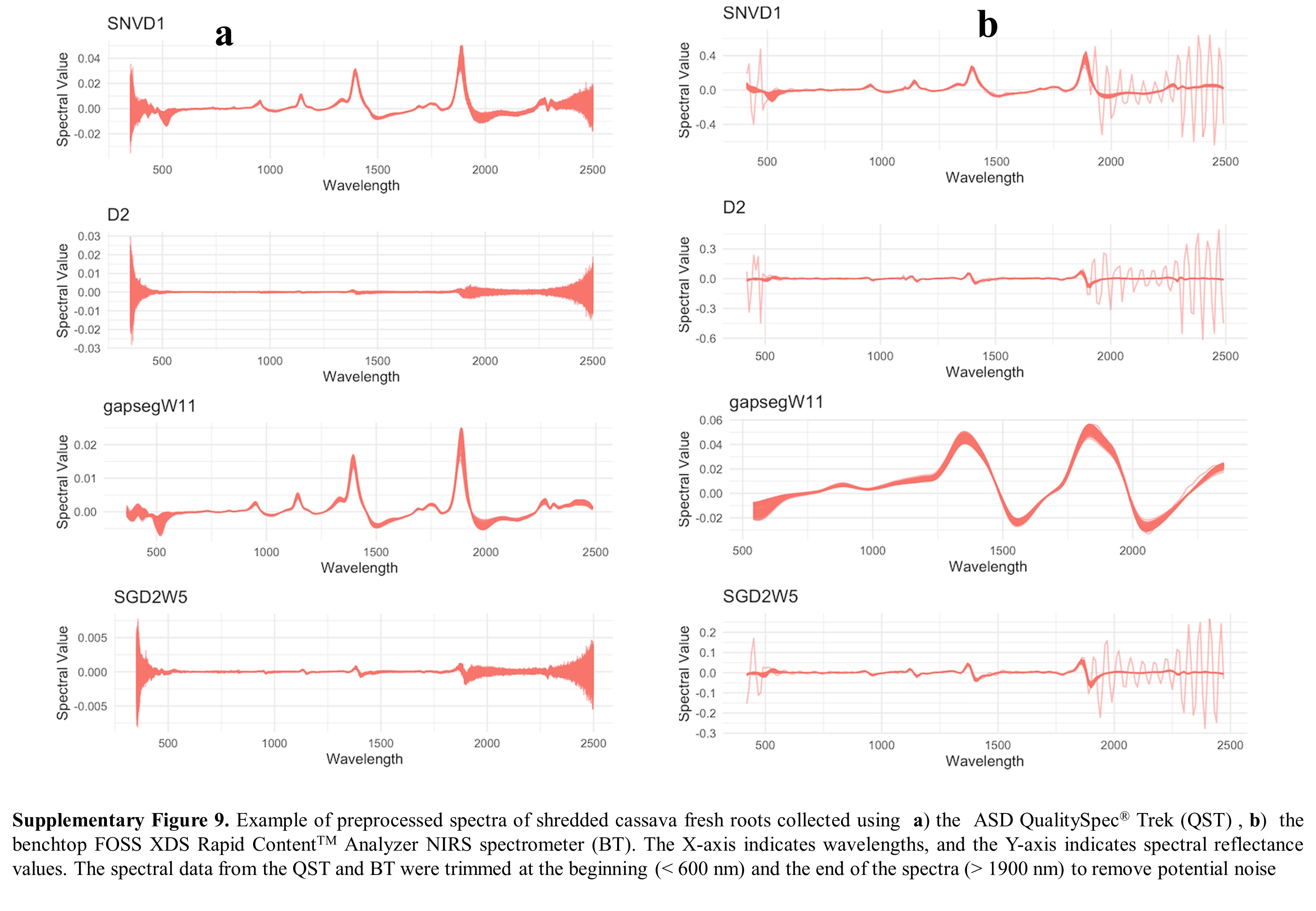

Supplement: Supplementary file 11 [file Image_9.jpeg]

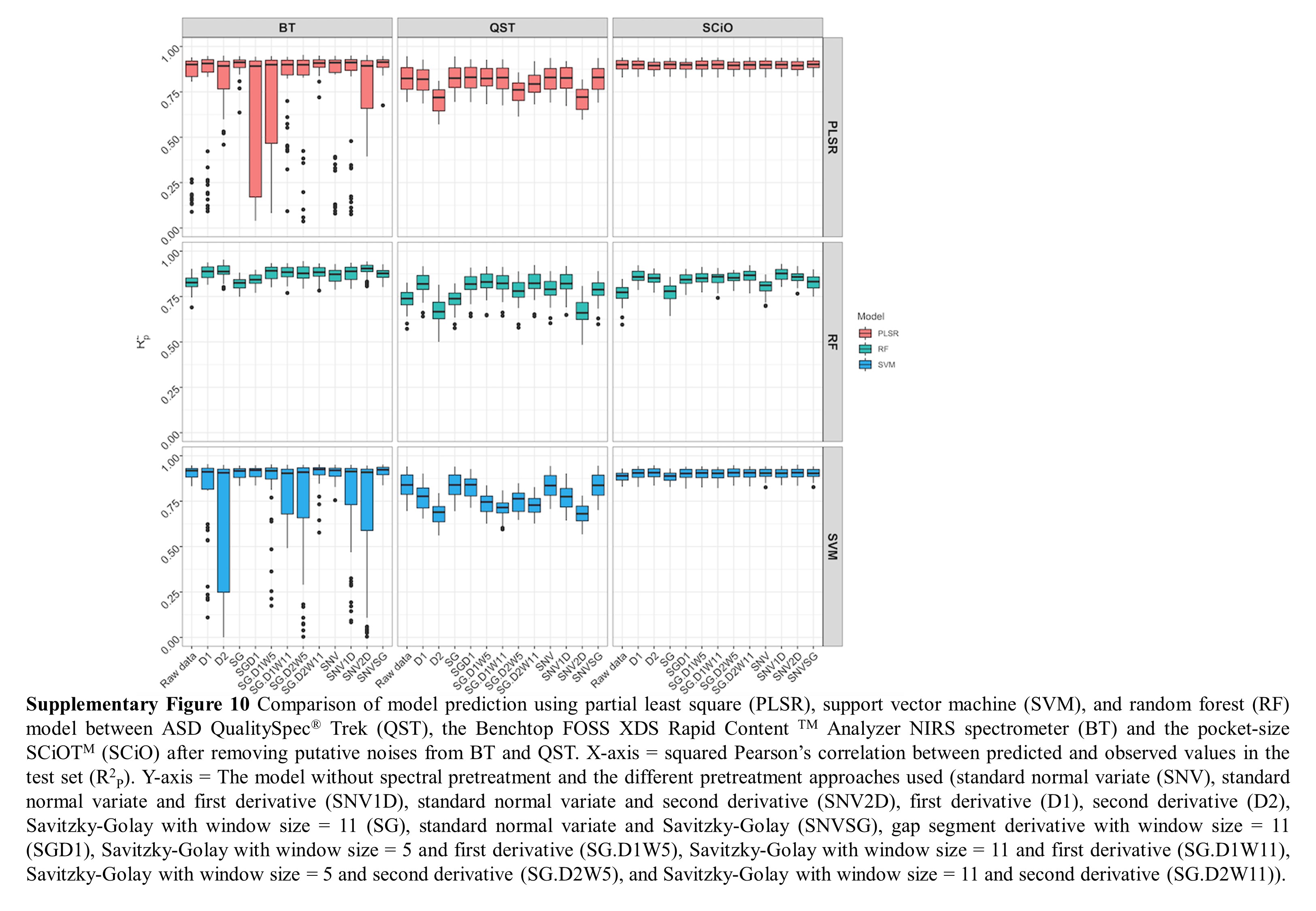

Supplement: Supplementary file 12 [file Image_10.jpeg]

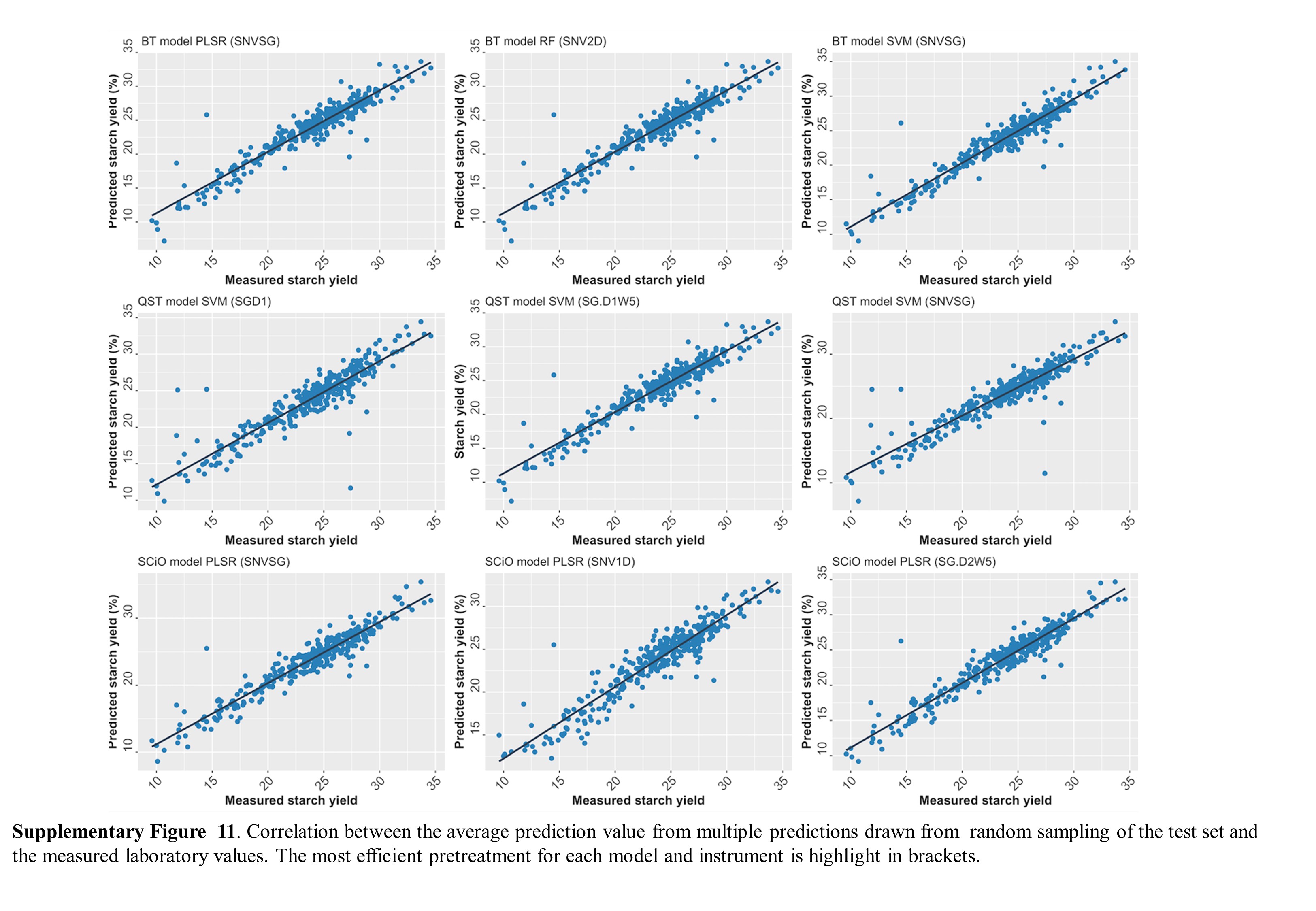

Supplement: Supplementary file 13 [file Image_11.jpeg]
